# Human occupation of the Afroalpine Bale Mountains at the onset of the African Humid Period

**DOI:** 10.1007/s10980-026-02337-8

**Published:** 2026-04-09

**Authors:** Götz Ossendorf, Minassie Girma Tekelemariam, Noora Taipale, Alexander R. Groos, Agazi Negash, Dries Cnuts, Naki Akçar, Christof Vockenhuber, Zinash Kefyalew Tariku, Trhas Hadush Kahsay, Veerle Rots, Ralf Vogelsang

**Affiliations:** 1 Human and Earth System Coupled Research (HESCOR), Institute of Prehistoric Archaeology, University of Cologne, Bernhard-Feilchenfeld-Straße 11, 50969 Cologne, Germany; 2 Department of Geography, Environmental Informatics, Philipps University of Marburg, Deutschhausstraße 10, 35032 Marburg, Germany; 3 Palaeolithic Research Unit, Institute of Prehistoric Archaeology, University of Cologne, Bernhard-Feilchenfeld-Straße 11, 50969 Cologne, Germany; 4 TraceoLab/Prehistory, University of Liège, Place du 20-Août 7 (Bât. A4), 4000 Liège, Belgium; 5 Fund for Scientific Research (F.R.S.-FNRS), Rue d’Egmont 5, 1000 Brussels, Belgium; 6 Institute of Geography, Friedrich-Alexander-Universität Erlangen-Nürnberg, Wetterkreuz 15, 91058 Erlangen, Germany; 7 Institute of Geography, University of Bern, Hallerstraße 12, 3012 Bern, Switzerland; 8 School of Earth Sciences, Addis Ababa University, PO Box 1176, Addis Ababa, Ethiopia; 9 Institute of Geology, University of Bern, Baltzerstraße 1+3, 3012 Bern, Switzerland; 10 Laboratory of Ion Beam Physics, ETH Zürich, Otto-Stern-Weg 5, 8093 Zurich, Switzerland; 11 Research Directorate, Ethiopian Heritage Authority, National Museum of Ethiopia, Yared Street, 2QQ6 7H5 Addis Ababa, Ethiopia; 12 Department of Geology and Geological Engineering, School of Earth Sciences and Engineering, Botswana International University of Science and Technology, Block 236, Palapye, Botswana

**Keywords:** African Humid Period, Glacial History, High-Altitude Adaptation, Obsidian Provenance, Lithic Technology, Use Wear, Residue Analysis

## Abstract

**Context:**

The reasons for the intermittent human use of harsh Afroalpine environments in prehistory remain unclear. High-resolution glacial and archaeological chronologies from Ethiopia’s Bale Mountains now offer insights into landscape change and human adaptations at high altitudes.

**Objectives:**

This study investigates the behavioral signatures of human occupation in Africa’s largest alpine environment around 15,000 years ago, focusing on local site use and integration into regional networks amid deglaciation and the abrupt onset of African Humid Period wet conditions.

**Methods:**

This research integrates surface exposure dating of moraine boulders and radiocarbon dating of archaeological rock shelter deposits with detailed analyses of lithic materials from three stratified sites in the Bale Mountains. We use multivariate statistical analyses of electron microprobe data to determine the geochemical provenance of obsidian artifacts. Lithic technological analysis is based on systematic recording of artifact attributes to reconstruct key stages of production. Functional analyses include use-wear and residue studies conducted using stereomicroscopy, reflected light microscopy, and scanning electron microscopy (SEM–EDX).

**Results:**

This study provides a detailed reconstruction of the final deglaciation phase in the Bale Mountains and identifies distinct patterns of lithic acquisition, production, and use across three contemporaneous sites. Dimtu, located on the formerly glaciated plateau and representing the highest known stratified archaeological site in Africa, is distinguished by a focus on the production of rare but specific pointed flakes. Simbero exhibits standardized backed tool production and evidence of hafting, while the Webi Gestro assemblage includes bladelets and notched tools; wear on unretouched bladelets indicates their use in transverse and longitudinal motions for processing activities and possibly as projectile elements. Geochemical results reveal obsidian exchange between high altitudes and lowlands, suggesting extensive social networks reinforced by technological and behavioral parallels.

**Conclusions:**

Human strategies at high altitudes closely mirror contemporaneous lowland behavior, revealing synchronous patterns across ecological zones. Similar patterns during other periods point to broader systemic dynamics. Conventional refugium-based explanations fail to fully capture these patterns, highlighting the need to examine diachronic shifts in the scale, connectivity, and intensity of prehistoric networks across ecozones.

**Supplementary Information:**

The online version contains supplementary material available at 10.1007/s10980-026-02337-8.

## Introduction

### High-altitude archaeology in Ethiopia

In global archaeological discourse, landscapes situated above roughly 2,500 m above sea level (m asl) are generally – though not always consistently – classified as high-altitude environments (Aldenderfer 1998). At these elevations, hypoxia poses a substantial physiological stressor, especially for populations originating from lower altitudes (Moore 2001; Beall 2007; West 2012). For prehistoric groups seeking to establish permanent settlements in such settings, cultural and technological adaptations were not optional but essential for long-term survival (Aldenderfer 2019). Beyond hypoxia, various high-altitude environments pose a combination of additional challenges, such as low temperatures, high ultraviolet radiation, increased water loss, elevated basal metabolic demands, and limited resource productivity (Moore 2001; Burtscher et al. 2018). While not all these stressors are altitude-specific (Körner 2007), their combined effect makes life at altitude particularly and variably demanding. Over the past decade, an expanding body of interdisciplinary research has examined both the nature of these challenges of global high-altitude and mountain occupations and the diverse human strategies developed to mitigate them (e.g., Brantingham and Gao 2006; Capriles et al. 2016; Chen et al. 2014, 2019; Ge et al. 2024; Haas 2023; Meyer et al. 2017; Ossendorf et al. 2019; Pazan et al. 2022; Pitblado 2017; Rademaker et al. 2014; Shnaider et al. 2018; Stewart et al. 2012, 2016; Stirn 2014; Way et al. 2025; Xia et al. 2024; Zhang et al. 2018). Within Africa, Ethiopia stands out as the primary setting for high-altitude archaeology, encompassing the vast majority of the continent’s highlands – about 80% of Africa’s landmass above 3,000 m asl lies within its borders (Siebert and Ramdhani 2004). Consequently, Ethiopia, with its network of isolated high-altitude *sky islands* atop its subdivided highlands (Aldenderfer 2006; Assefa et al. 2007), possesses excellent conditions for advancing our knowledge. Research in this field is still in its early stages, but initial evidence suggests a deep-time human presence. Though undated, Acheulean handaxes from Mount Dendi at 3,000 m asl likely predate the emergence of *Homo sapiens* and form the oldest African evidence for hominin activity in high altitudes (Vogelsang et al. 2018, 2023). Strikingly, several well-known early Acheulean sites up to 1.6 million years old – Gadeb (Clark and Kurashina 1979; de la Torre 2011), Fanta (Lanzarone et al. 2016), Melka Kunture (Gallotti and Mussi 2017; Mussi 2023), and Melka Wakene (Hovers et al. 2021; Gossa et al. 2023) – are located just below 2,500 m asl. This distribution may reflect a putative *hypoxia threshold* limiting hominin dispersal, but further investigation and validation are needed. Yet clear evidence for sustained high-altitude settlement appears only late within the Middle Stone Age (MSA), a period associated with anatomically modern humans (Richter et al. 2017). The site of Fincha Habera in the Ethiopian Bale Mountains provides the earliest and most compelling example. Occupied repeatedly between 47 and 31 thousand years calibrated before present (ka cal. BP), it demonstrates not only long-term residence but also targeted exploitation of Afroalpine resources (Ossendorf et al. 2019). Subsistence at the site centered on the endemic fossorial giant root-rat (*Tachyoryctes macrocephalus*), which remained a dietary staple for millennia. This occupation occurred during the *local* Last Glacial Maximum (LGM; Groos et al. 2021a), underscoring human resilience under extreme climatic stress. Artifacts further attest to long-distance interactions across ecological zones, including lowland deserts and savannas (Ossendorf et al. 2023). Broadly coeval sites are known to be located just below 2,500 m asl, such as Mochena Borago (Brandt et al. 2012, 2017), but also in even lower elevations and distinct ecozones, such as Goda Buticha (Tribolo et al. 2017), Porc Épic (Assefa 2006), Gorgora (Sahle et al. 2024), Deka Wede 1 (Ménard et al. 2014), Gotera 10 (Fusco et al. 2025), or HAL-A27 (Niespolo et al. 2021). These findings challenge the view that high-altitude landscapes were used as environmental refugia during periods of adverse climatic conditions in the surrounding lowlands. Although refugia concepts remain influential (e.g., Ambrose 1998; Basell 2008, 2013; Beyin 2011; Blinkhorn et al. 2022; Blinkhorn and Grove 2018; Brandt et al. 2012, 2017; Fischer et al. 2020, 2021; Foerster et al. 2012; Hildebrand et al. 2010, 2019; Jones et al. 2018; Khalidi et al. 2018, 2020; Leplongeon et al. 2020a, b, 2025; Ménard and Bon 2015; Sahle 2020; Timbrell et al. 2022; Tribolo et al. 2017; Viehberg et al. 2018), they are difficult to test, let alone by diverging understandings on the respective spatial and temporal scales or environmental details. Moreover, the historical concentration of archaeological research in Ethiopia’s lowlands – especially the Main Ethiopian Rift (MER), the Afar Depression, and the Omo Valley – has left large parts of its highlands virtually unexplored (Fernández et al. 2007; Sahle 2020). This imbalance reinforces older narratives portraying the highlands as marginal, impassable, and unattractive for prehistoric settlement. Against this background, it is essential to investigate the contexts of repeated – yet discontinuous – high-altitude occupations in order to approach the motivations and strategies behind human engagement with such harsh environments. In this study, we examine an additional phase of Late Pleistocene settlement in the Bale Mountains, dating to the onset of the early African Humid Period (AHP; de Menocal 2000), around 15–14 ka cal. BP. This work draws on interdisciplinary archaeological, paleoecological and paleoclimatic research conducted by an Ethio-European Research Unit (DFG RU2358), which investigated the dynamics between landscape transformation and human settlement in Africa’s largest high-altitude alpine environment. We begin by reviewing regional climate conditions and examining local environmental changes in the Bale Mountains in light of new glacial chronological data. We then present and analyze the lithic assemblages, situating them within the dynamic human behavioral processes that underlie their static archaeological context, in order to explore patterns of human high-altitude use. Finally, we assess the wider archaeological and paleoenvironmental evidence to evaluate whether this high-altitude setting may have functioned as an environmental refugium.

### The study area: Bale Mountains (south-central Ethiopia)

#### Physical setting and ecological context

**Fig. 1:**
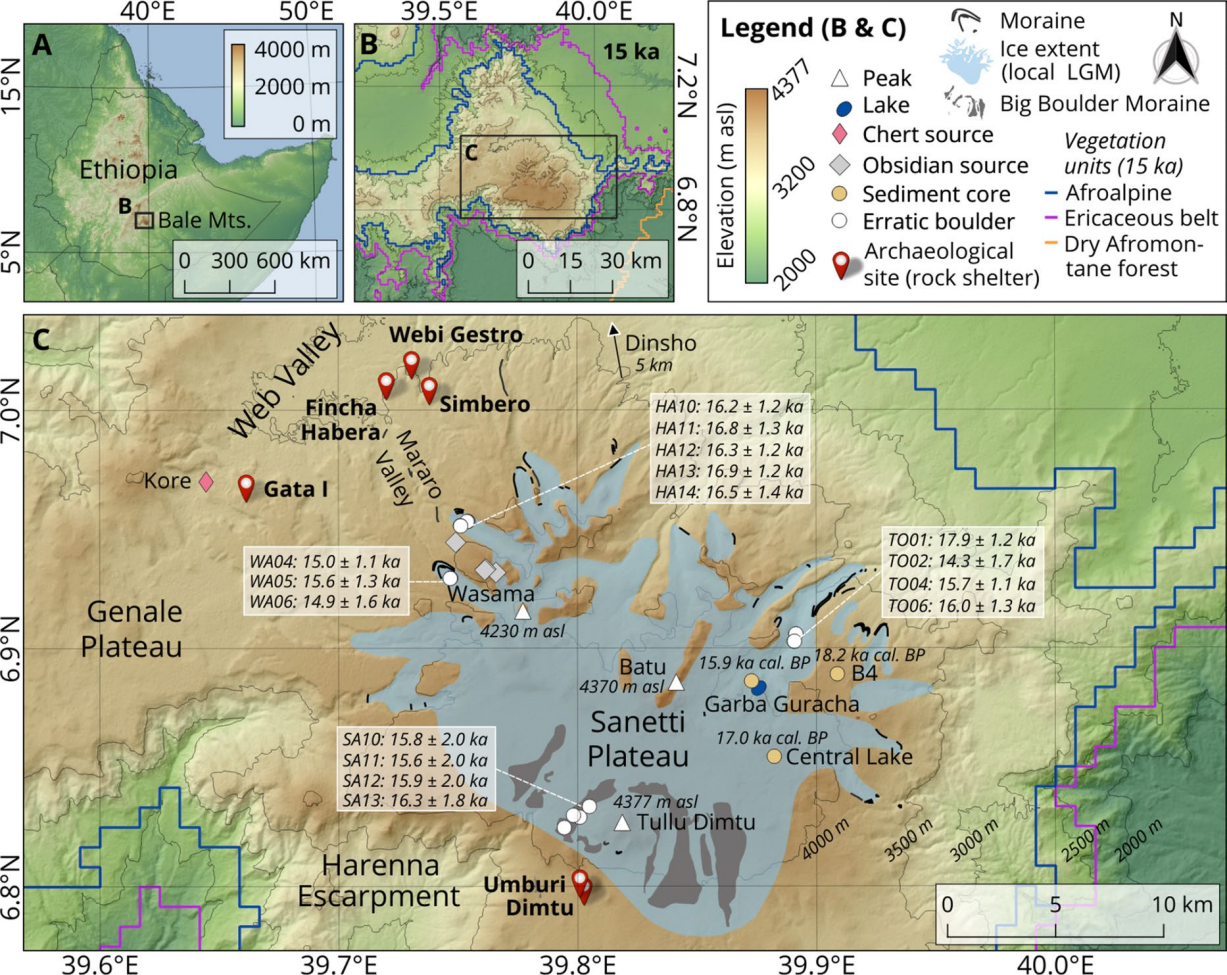
Maps of the Bale Mountains region illustrating key archaeological and environmental archives discussed in the text. **A** Location of the Bale Mountains within Ethiopia and the Horn of Africa. **B** Approximate distribution of major Afroalpine and Afromontane vegetation units at 15 ka cal. BP in the Bale Mountains, based on palaeoenvironmental reconstructions by Casas-Gallego et al. (2023). **C** Maximum ice extent during the *local* Last Glacial Maximum (42–28 ka), with cosmogenic ^36^Cl surface exposure ages from the innermost moraines in the Wasama (WA), Harcha (HA), and Togona (TO) valleys (Groos et al. 2021a), as well as new.^3^⁶Cl ages from the inner Big Boulder Moraine on the Sanetti Plateau (SA), indicating the onset of the final deglaciation phase. Also shown are the locations of sediment cores from the B4 glacial depression, Central Lake, and Lake Garba Guracha, annotated with the respective onset of sedimentation (Bittner et al. 2020; Mekonnen et al. 2022; Chernet et al. 2025). Archaeological rock shelters and local sources of lithic raw materials (chert, obsidian) include the newly introduced sites Dimtu, Simbero, Webi Gestro, Gata I, Umburi, and Kore, apart from Fincha Habera and Wasama, described in Ossendorf et al. (2019, 2023)

The Bale Mountains are located approximately 400 km southeast of Addis Ababa, the capital of Ethiopia (Fig. 1A). Geographically, they belong to the Bale-Arsi massif, which forms the western part of the Southeastern Ethiopian Highlands (Miehe and Miehe 1994). Its central high plateau, the Sanetti Plateau, is home to Mount Tullu Dimtu (4,377 m asl), the second-highest peak in Ethiopia (Fig. 1C; Hillman 1988). To the south and southeast, the plateau is bordered by the steep Harenna Escarpment. In contrast, the north is characterized by high ridges and broad valleys that gradually merge into the extensive Bale-Arsi Plateau and further into the lowlands of the MER (Tiercelin et al. 2008). The Bale Mountains constitute the largest continuous area above 3,000 m asl in Africa (Groos et al. 2021a), hosting the most extensive Afroalpine and sub-Afroalpine vegetation belts on the continent (Miehe and Miehe 1994) (Fig. 1B). The climate of the Bale Mountains is primarily influenced by the annual migration of the Intertropical Convergence Zone (ITCZ), which shifts between 10° N and 10° S. Today, the region experiences a long rainy season from March to October and a short dry season from November to February (Seleshi and Zanke 2004; Levin et al. 2009; Costa et al. 2014). Precipitation patterns are bimodal, with a central peak occurring from July to October, and a second peak from March to June (Kidane et al. 2012). Moist air masses predominantly originate from the Indian Ocean, while northerly winds dominate during the dry season (Lemma et al. 2020; Stojanovic et al. 2022). Orographic effects enhance precipitation in this area compared to other regions at similar latitudes (Kebede and Travi 2012). For instance, 1,000–1,500 mm of precipitation falls along the Harenna Escarpment, whereas the northern declivity receives only 800–1,000 mm per year (Miehe and Miehe 1994; Umer et al. 2007). Temperatures at Dinsho (3,170 m asl; Fig. 1C) range from an average minimum of 0.6 °C to a maximum of 11.8 °C, with nocturnal frost frequently occurring in the higher areas during the dry season (Tiercelin et al. 2008; Groos et al. 2022). Vegetation in the Bale Mountains is distinctly structured based on elevation and precipitation. Unlike in the past (Fig. 1B), the Afromontane Forest belt today ranges from approximately 1,400 to 3,300 m and is dominated in the south by species such as *Podocarpus gracilior*, *Syzygium guineense*, and *Aningeria adolfi-friederici*, while the northern slopes are characterized by *Juniperus procera*, *Hagenia abyssinica*, and *Hypericum revolutum* (Friis 1986; Bussmann 1997). Above this forest belt lies the Ericaceous belt, which spans approximately 90,000 hectares between 3,000 and 3,800 m asl and is dominated by *Erica trimera*, apart from *Erica arborea* (Miehe and Miehe 1994; Hailemariam et al. 2016). The Afroalpine belt begins at elevations above 3,800 m asl, marked by open vegetation rich in tussock grasses and dominated by *Helichrysum splendidum*, *Alchemilla haumannii*, and giant lobelia (*Lobelia rhynchopetalum*) (Yineger et al. 2008). Due to their vast size, isolation, and climatic history, the Bale Mountains also support an exceptionally rich endemic fauna, contributing to the region as a unique center of biodiversity (Hillman 1988; Yalden and Largen 1992; Williams et al. 2004). In addition to numerous rare and endemic bird and amphibian species, iconic mammals such as the Ethiopian wolf (*Canis simensis*), mountain nyala (*Tragelaphus buxtoni*), Menelik’s bushbuck (*Tragelaphus scriptus meneliki*), Bale vervet (*Chlorocebus djamdjamensis*), and the giant root-rat (*Tachyoryctes macrocephalus*) are characteristic of the region.

#### Late Glacial climate change and Afroalpine landscape transformation

Between approximately 22 and 20 ka, Last Glacial Maximum (LGM) conditions prevailed in the Horn of Africa: significantly colder than today’s temperatures and very dry (Gasse 2000; Tierney et al. 2011). Lake sediments show considerably reduced water levels, coarse-grained deposits, and evaporites (Chalié & Gasse 2002; Foerster et al. 2012; Lamb et al. 2007; Umer et al. 2007). High dust concentrations in marine sediments indicate sparse vegetation and severe wind erosion (deMenocal et al. 2000). Isotope analyses s(δ^1^⁸O, δD) and pollen profiles document low precipitation, intense evaporation, and the dominance of steppe and desert plants (Lamb et al. 2007; Umer et al. 2007; Foerster et al. 2012). Conditions also remained predominantly dry and cold during the Late Glacial and Heinrich Stadial 1 (approx. 18–15 ka) (deMenocal et al. 2000, 2022; Arz et al. 2003; Marshall et al. 2011; Foerster et al. 2012; Tiercelin et al. 2008). Between approximately 15–13 ka (roughly correlating with the Bølling-Allerød Interstadial), a first distinct wet phase began in the Horn of Africa, referred to here as the *early* AHP (deMenocal et al. 2000; Barker et al. 2004; Foerster et al. 2022). This phase of abrupt increased humidity characterizes large parts of northern and tropical Africa after the global LGM (deMenocal et al. 2000; Tierney & deMenocal 2013). It was primarily driven by orbital precession, which enhanced summer insolation over northeast Africa. Temperatures were above global LGM levels but still cooler (~ 2–3 °C) than in the Holocene (Tierney & deMenocal 2013). Proxy data show rising lake levels, finer sediments, decreasing dust concentrations, and increasing vegetation density (Foerster et al. 2022; Lamb et al. 2007; Umer et al. 2007). Pollen profiles from the Ethiopian Highlands confirm the spread of wetlands (Umer et al. 2007; Tiercelin et al. 2008). Although local environmental records remain notably sparse in the Horn of Africa (Lesur et al. 2016), they are essential for reconstructing climate and ecosystem dynamics at spatial scales relevant to past human populations, and for determining whether broader climatic trends are locally reflected (Hildebrand et al. 2019). During the *local* LGM, which occurred already at approximately 42–28 ka, glaciers covered about 265 km^2^ of the Bale Mountains. An ice cap extended from the central Sanetti Plateau (Fig. 1C) into the western, northern and eastern valleys (Groos et al. 2021a). This glaciation coincided with a cooling of 4.4–6.0 °C, causing Afroalpine vegetation to descend to lower elevations. In the Wasama, Harcha, and Togona valleys (Fig. 1C), well-preserved moraine sequences record post-LGM glacier culminations between 19 and 17 ka. Glacial recession began between 18 and 17 ka. The innermost moraines indicate a glacier stillstand around 16 ka, which marks the onset of the final deglaciation phase in the Bale Mountains (Groos et al. 2021a). However, while the glacial chronology of the valleys is relatively well constrained, the deglaciation history of the Sanetti Plateau and its relationship to the Afroalpine landscape transformation remains unresolved. Clear evidence of increased humidity has been recorded in all sediment archives of the Bale Mountains beginning around 15 ka cal. BP (Fig. 1C; Gil-Romera et al. 2019; Mekonnen et al. 2022; Chernet et al. 2025). The onset of sedimentation on the Sanetti Plateau occurred at approximately 18.2 ka (B4) and 17.0 ka (Central Lake), and slightly later at Lake Garba Guracha in the upper Togona Valley (15.9 ka cal. BP) (Fig. 1C; Bittner et al. 2020; Mekonnen et al. 2022; Chernet et al. 2025). During this period, herbaceous and endemic high-altitude plant species expanded throughout the Afroalpine belt. Pollen and sedaDNA analyses indicate an increase in species diversity among grasses, herbs, and shrubs. Despite this diversification, vegetation remained mosaic-like due to the extreme altitudinal gradients (Gil-Romera et al. 2019; Mekonnen et al. 2022). In the subalpine zone, *Erica* shrubs expanded into areas that were previously dry, forming denser shrub assemblages alongside increased cover of mosses and lichens. Natural fire regimes influenced vegetation structure, although the ecosystem remained generally fire-resistant (Gil-Romera et al. 2019).

### Chronostratigraphy of early African Humid Period archaeological sites in the Bale Mountains

**Fig. 2:**
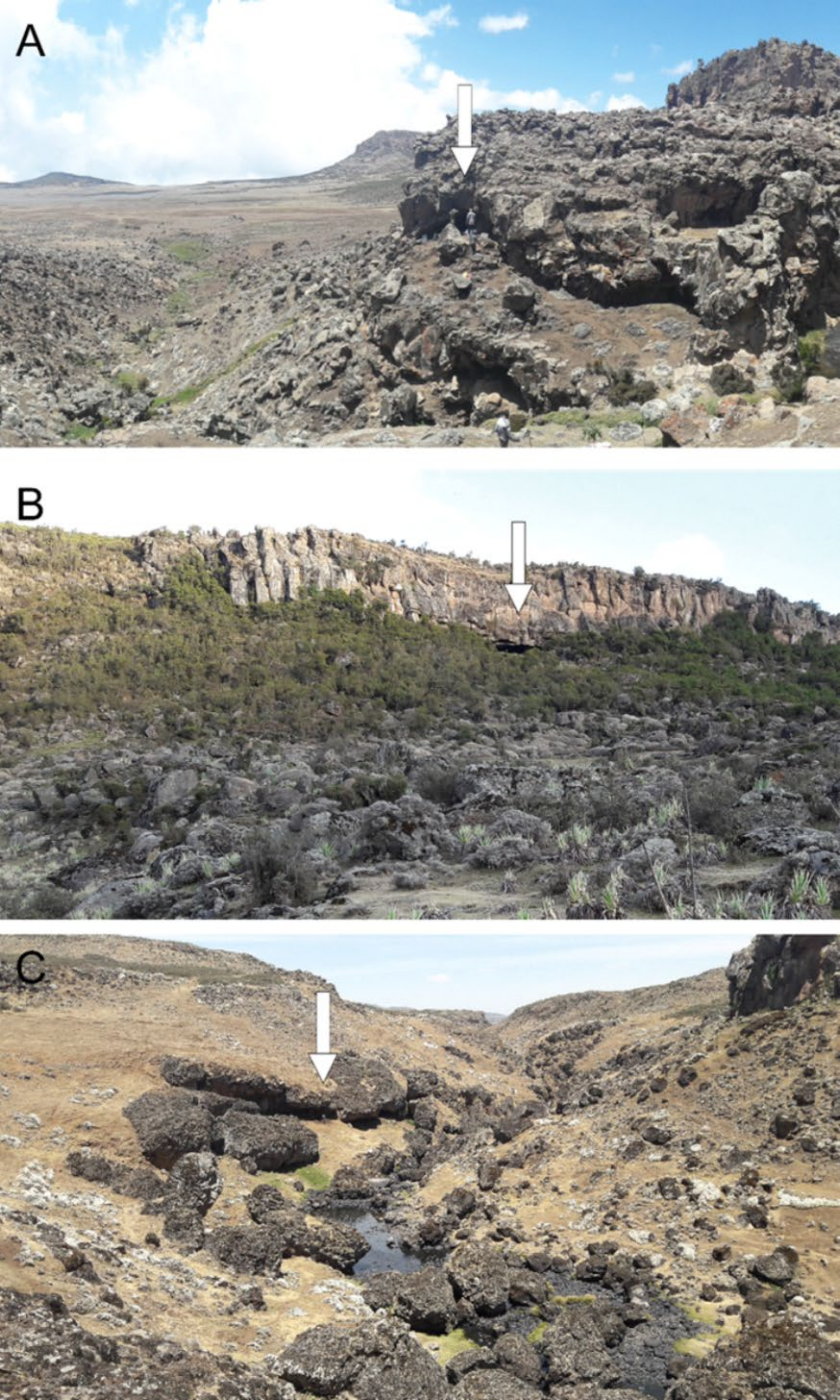
Archaeological sites (white arrows) with early AHP-dated sequences in the Bale Mountains and their immediate surroundings.** A** Dimtu rock shelter on the central Sanetti Plateau. **B** Simbero rock shelter in the *Erica*-covered cliffs of the eponymous valley. **C** Webi Gestro on the leeward side of a rock wall next to the eponymous river gorge in the upper Web Valley. Site locations are indicated in Fig. 1C. Photographs were taken at the end of the dry season and do not reflect the region’s prevailing humid conditions

Systematic archaeological surveys and test excavations conducted at elevations between 3,400 and 4,200 m asl in the Bale Mountains have led to the identification of more than 100 archaeological sites. Among these, a small number of lithic surface scatters are directly associated with primary raw material sources (N = 8), including obsidian outcrops on the southern slopes of the Wasama Ridge and several closely spaced chert outcrops at Kore (Fig. 1C). Another group comprises lithic surface finds (N = 28), predominantly located on rocky slopes in proximity to rock shelters. The majority of the identified sites, however, are stratified deposits found within rock shelters (N = 72). Notably, a dense cluster of these shelter sites is concentrated along the northwestern escarpment of the Bale Mountains (n = 50), reflecting the geomorphological abundance of rock shelters in this area (Reber et al. 2018). Additional shelter sites were documented on the central Sanetti Plateau (n = 14) and in riverine contexts along the northern slopes (n = 6), while only a few shelter sites were recorded on the steep, forested southern Harenna Escarpment (n = 2) (Fig. 1C). Eleven rock shelter sites with stratified deposits were excavated and studied in more detail. Except for two sites with Middle Stone Age (MSA) deposits, most contain human occupations in post-glacial sediments dating maximally from 15 ka cal. BP to the present. The youngest deposits consist mainly of decomposed dung mixed with loamy sediments and single *Erica* posts, likely indicating former hut remains (Reber et al. 2018). These layers, associated with very little archaeological material, date to the last 1,000 years and sometimes cut deep into lower deposits (Tekelemariam 2021). Earlier lithic assemblages attributed to hunter-gatherer groups – often accompanied by faunal remains and diverse artifact types – are frequently found in deeper stratigraphic layers. These assemblages are commonly associated with substantial charcoal accumulations dated to the Late and Middle Holocene. Early Holocene dating results are rare and associated with sparse and undiagnostic lithics. In contrast, deposits from the early African Humid Period have been found in the bottommost layers of three sites (see Fig. 2), which will be introduced in the following.

**Fig. 3:**
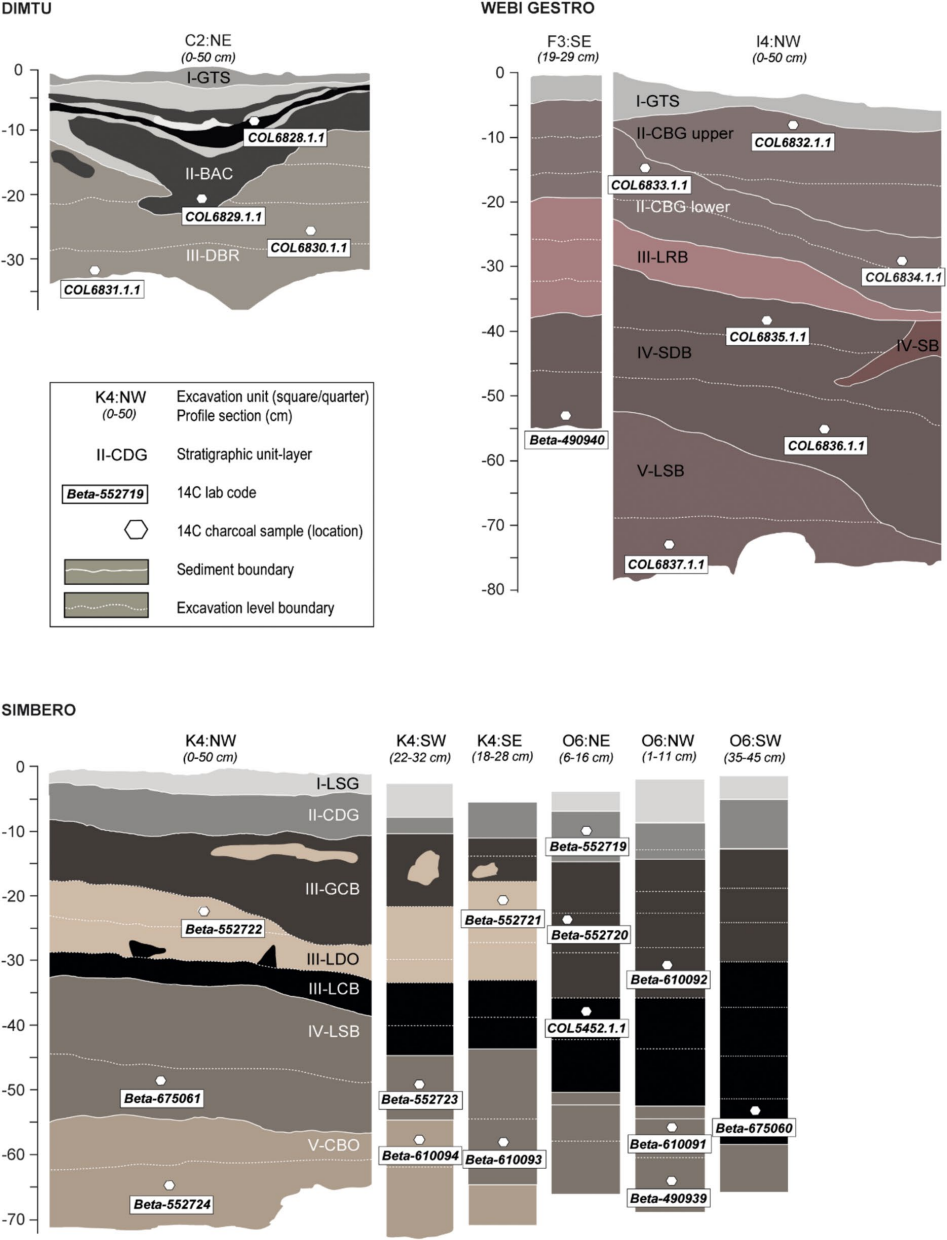
Selected stratigraphic profiles at Dimtu (top left), Simbero (bottom), and Webi Gestro (top right). At all sites, well-defined early AHP layers occur at the base of the stratigraphic sequences, directly overlying bedrock. Positions and designations of radiocarbon-dated charcoal samples from all layers are indicated. The legend within the box denotes the principal symbols and labels. For detailed stratigraphic information, see the Supplementary Materials and Table 1; for a summary of radiocarbon results, see Table S1. Vertical measurements are provided in cm relative to the respective surface

**Dimtu** rock shelter (6.80°N, 39.80°E; 4,023 m asl), located ~ 2 km from Tullu Dimtu on the central Sanetti Plateau of the Bale Mountains (Fig. 1C), is a previously unreported high-altitude site embedded in a weathered basalt ridge (Fig. 2) naturally sheltered from strong trade winds. Test excavations of a single 1 m^2^ area revealed two stratigraphically distinct lithic assemblages preserved in predominantly dry, autochthonous sediments with minimal post-depositional disturbance (Fig. 3**, **Table 1). The lower assemblage (SU III, Layer DBR) from a sandy matrix with charcoal dates to the Late Glacial (14,879–13,861 cal. BP), while the upper assemblage (SU II, Layer BAC) within a banded ash and charcoal layer reflects a Late Holocene occupation (Table S1). To our knowledge, Dimtu represents the highest stratified archaeological site in Africa, demonstrating episodic human use of high-altitude environments, only exceeded by lithic surface scatters at the Wasama Ridge obsidian outcrops (> 4,200 m asl; Ossendorf et al. 2019) that document repeated prehistoric activity.

**Table 1:** Stratigraphic overview of the Bale Mountains archaeological sites (Dimtu, Simbero, Webi Gestro). Stratigraphic units (SUs) are listed from youngest to oldest within each site. Sediment characteristics, human activity, taphonomic indicators, and radiocarbon-based age ranges are summarized. Detailed stratigraphic descriptions are provided in the Supplementary Materials, full chronological data in Table S1, and selected stratigraphic profiles in Fig. 3

Site	Stratigraphic unit – sediment characteristics	Human activity & taphonomic indicators	Age range
Dimtu (Sanetti Plateau rock shelter, 4023 m asl)	SU I (GTS) – thin aeolian silty sand surface layer with angular clasts	Largely sterile; minor roof fall and root intrusions	Undated (recent surface deposit)
	SU II (BAC) – well-sorted silt with laminated ash and charcoal horizons	Hearths, burnt sediments and trampling; charcoal, bones and lithic artifacts indicating repeated occupation	1.7–1.5 ka cal BP
	SU III (DBR) – poorly sorted slightly gravelly sand resting on bedrock	Charcoal, ash, bones and lithics evenly distributed; fire use and trampling; minor carbonate crust formation	14.9–13.8 ka cal BP
Simbero (northwestern escarpment rock shelter, 3520 m asl)	SU I (LSG) – silty–sandy surface sediment with organic inputs	Sparse artifacts and hearth traces; strong biological disturbance (roots, dung, organic debris)	Undated (recent surface deposit)
	SU II (SCG) – compact sandy sediment strongly affected by decomposed dung	Dung accumulations and phosphate crusts; few bones and artifacts (incl. beads and lithics)	Modern (radiocarbon)
	SU III (GCB–LDO–LCB) – clayey to silty sediments reflecting complex deposition under moist conditions	Abundant charcoal, bones and lithic artifacts; hearths, burnt sediments and trampling; localized carbonate crusts	3.8–2.2 ka cal BP
	SU IV (LSB) – well-sorted sandy sediment with ash and charcoal fragments	Evidence of fire use and trampling; bones and lithics indicating repeated occupation	8.2–5.1 ka cal BP
	SU V (CBO) – silty–sandy basal deposit with angular clasts	Bones and lithic artifacts; evidence of fire use and occupation surfaces	15.0–14.3 ka cal BP
Webi Gestro (river-margin escarpment site, 3423 m asl)	SU I (GTS) – moist silty surface deposit with mixed fabric	Sparse charcoal; minor burning and trampling without clear hearths	Undated (recent surface deposit)
	SU II (CBG) – compact sandy sediment with abundant charcoal and ash	Hearths, burnt sediments and trampling; bones and lithic artifacts documenting repeated occupation	Recent / undated (upper); 6.5–6.3 ka cal BP (lower)
	SU III (LRB) – well-sorted clayey silt with low clast content	Very low artifact density; minimal charcoal and ash; phase of reduced site use	6.6–6.4 ka cal BP
	SU IV (SDB–SB) – sandy occupation deposits with angular clasts	Abundant charcoal, ash, bones and lithics; hearths, burnt sediments and trampling indicators	14.8–13.9 ka cal BP
	SU V (LSB) – sandy basal deposit with roof fall	Bones and lithics present; hearth evidence ambiguous; episodic occupation	14.8–14.0 ka cal BP

**Simbero** rock shelter (7.01°N, 39.74°E; 3,520 m asl) was first reported as “Rock Shelter 1” by Reber et al. (2018) and has since been studied in detail: Tekelemariam (2021) analyzed its lithic assemblages, while Tariku (2021) examined the faunal remains. Ongoing sedimentological and soil biogeochemical studies further reinforce Simbero’s value as a reference stratigraphy for understanding human–environment interactions in the Bale Mountains. The site is located ~ 50 m above the Simbero Valley on the northwestern escarpment, overlooking the upper Web Valley (Fig. 1C**, **Fig. 2). Test excavations over two 2 m^2^ squares, spaced approximately 5 m apart, revealed a dense, diverse, and stratified sequence reflecting discontinuous but recurrent occupations from the Late Glacial through the Mid- and Late Holocene into the recent past. Persistent humid conditions and strong anthropogenic impacts – including dung accumulation, burning, trampling, charcoal accumulation, dense cultural materials, and repeated reworking of occupation surfaces – characterize the stratigraphy (Table 1). Lithic and faunal assemblages are particularly abundant in the Late Holocene layers, with ochre, pottery, and grinding stones appearing regularly. The lowermost layer, SU V (Fig. 3), is dated to the early AHP, with two independent radiocarbon results assigning its associated lithic assemblage between 14,958 and 14,262 cal. BP (Table S1).

**Webi Gestro** (7.02°N, 39.73°E; 3,423 m asl) is not a rock shelter in the strict sense, but an overhanging basalt wall running parallel to the small Webi Gestro stream on the northwestern escarpment of the Bale Mountains (Fig. 1C**, **Fig. 2). Beneath the wall, a narrow terrace formed above the stream is followed by a steep slope of ~ 15 m width descending to the water. Excavations were carried out directly beside the basalt wall in two test squares set 6 m apart. Both revealed an identical stratigraphy, with a moist, loamy, reddish sedimentary sequence accumulated largely in situ (Table 1), with good organic preservation but limited faunal remains. Three distinct charcoal concentrations are preserved interbedded within the sediment matrix. Overall, Webi Gestro records episodic, short-term human presence within a fluvially influenced but stratigraphically stable depositional setting: radiocarbon dating of the charcoal layers and their associated cultural material documents Late Glacial activity during the early African Humid Period (Table S1**:** bottommost SU IV–V; four dates between 14,836–13,882 cal. BP), Mid-Holocene use (SU II lower and SU III), and very recent occupation (SU II upper).

In order to capture the full range of uncertainty, early AHP-related radiocarbon dates were calibrated separately with IntCal20 and SHCal20 here (Table 2), as fixed mixed calibrations are inappropriate in eastern Africa due to seasonal ITCZ-driven ^14^C variability (Marsh et al. 2018; Hogg et al. 2020). At least seven of the eight radiocarbon determinations from the respective bottommost deposits at Dimtu, Simbero, and Webi Gestro consistently overlap, regardless of the selected confidence interval or calibration curve. At the 95.4% probability level, the complete set constrains the period of occupation to a maximum span of 1,097 years (1,104 years using SHCal). A subset of six dates indicates a minimum duration of 483 years (542 with SHCal). Taken together, all three archaeological sites offer a consistent estimate for the length of early AHP-related human presence in the Bale Mountains. This occupation phase is followed by a period for which no radiocarbon dates are currently available, lasting until sporadic Early Holocene occupations at Gata I (10,177–9,820 cal. BP) and Umburi (9,011–8,770 cal. BP), and continuing into the Mid-Holocene at Simbero (Table S1**;**Fig. 1C).

**Table 2:** AMS radiocarbon age determinations of charcoal samples from early African Humid Period (AHP) deposits in the investigated rock shelters. Results are given as 1σ and 2σ probability calendar year ranges, calculated with OxCal v4.4 (Bronk Ramsey 2009), using both IntCal20 (Reimer et al. 2020) and SHCal20 (Hogg et al. 2020) calibration curves. See Table 1 for stratigraphic summaries, Fig. 3 for stratigraphic provenance of the samples and Table S1 for non-early AHP-related additional radiocarbon dating results from the three sites studied

Site	Excavation unit	Strat. unit	Lab No	14C Age BP	Cal. BP (68.3%; IntCal)	Cal. BP (95.4%; IntCal)	Cal. BP (68.3%; SHCal)	Cal. BP (95.4%; SHCal)
Dimtu	C2:SW:L8	III-DBR	COL6830.1.1	12,181 ± 57	14,165–14,030	14,315–13,861	14,117–13,872	14,186–13,806
Dimtu	C2:SW:L10	III-DBR	COL6831.1.1	12,393 ± 58	14,822–14,295	14,879–14,180	14,800–14,191	14,845–14,108
Simbero	K4:SW:L9	V-CBO	Beta-610094	12,400 ± 30	14,807–14,323	14,845–14,262	14,795–14,239	14,831–14,171
Simbero	K4:NW:L10	V-CBO	Beta-552724	12,460 ± 40	14,900–14,472	14,958–14,322	14,831–14,333	14,910–14,276
Webi Gestro	I4:NE:L8	IV-SDB	COL6835.1.1	12,211 ± 58	14,193–14,047	14,780–13,882	14,182–14,026	14,323–13,815
Webi Gestro	I4:NE:L15	V-LSB	COL6837.1.1	12,256 ± 61	14,275–14,075	14,805–14,040	14,227–14,048	14,802–13,873
Webi Gestro	I4:NE:L12	IV-SDB	COL6836.1.1	12,531 ± 61	14,798–14,182	14,842–14,103	14,791–14,105	14,830–14,072
Webi Gestro	F3:SW:L10	IV-SDB	Beta-490940	12,370 ± 40	14,799–14,251	14,836–14,175	14,792–14,163	14,821–14,103

## Materials and methods

### Surface exposure dating of moraine boulders

To constrain the ice extent and onset of the final deglaciation phase on the central Sanetti Plateau, near the Dimtu rock shelter, and to assess potential local environmental and climatic changes at the onset of the early African Humid Period, we sampled four glacial erratics (Table 3) from the inner Big Boulder Moraine west of Tullu Dimtu (Fig. 1C**)** for ^36^Cl surface exposure dating. Exposure dating with the two most commonly applied cosmogenic nuclides, ^10^Be and ^26^Al, was not feasible because the volcanic rocks in the study area lack sufficient quartz, the required target mineral. As with previously dated boulders from nearby valleys (Fig. 1C) and other sites on the Sanetti Plateau (Groos et al. 2021a, 2021b), sample material was taken from the upper 5 cm of each boulder (SA10-13) using a hammer, angle grinder, and chisel. Topographic shielding was measured in the field with an inclinometer, although its effect on ^36^Cl production rates is negligible on the plateau. The four rock samples were crushed, sieved, leached, dissolved, and further chemically processed in the Surface Exposure Dating Laboratory at the University of Bern to remove sulphur (S) and isolate chlorine (Cl). At the 6 MV accelerator mass spectrometry (AMS) facility at ETH Zurich, residual ^36^S was eliminated with a gas-filled magnet (Vockenhuber et al. 2019), and total Cl and ^36^Cl concentrations were measured from one target following the isotope dilution method of Ivy-Ochs et al. (2004). Major and trace element concentrations, required for calculating element-dependent production rates, were measured on 10 g aliquots at Activation Laboratories Ltd. in Ontario, Canada (Table S2). Exposure ages were then derived from the measured Cl and ^36^Cl concentrations in combination with the major and trace element data. Calculations employed version 2.1 of the CRONUS Earth Web Calculator (https://cronus.cosmogenicnuclides.rocks/2.1/html/cl/https://cronus.cosmogenicnuclides.rocks/2.1/html/cl/; Marrero et al. 2016) and the physics-based, time-dependent Lifton-Sato-Dunai scaling framework (Lifton et al. 2014), which accounts for nuclide-specific production rates as well as spatio-temporal variations in the geomagnetic and solar magnetic fields. For further details on the application of surface exposure dating to moraine boulders in the Bale Mountains, see Groos et al. (2021a).

**Table 3:** Description of the geographic location and characteristics of the sampled erratic boulders from the inner Big Boulder Moraine on the Sanetti Plateau in the Bale Mountains

Rock sample	Lithology	Latitude (°N)	Longitude (°E)	Elevation (m asl)	Boulder length (m)	Boulder width (m)	Boulder height (m)	Sample thickness (cm)	Shielding factor
SA10	Trachyte	6.82905	39.80079	4071	2.5	2.2	1.7	3.0	0.9967
SA11	Trachyte	6.83355	39.80481	4086	6.0	4.5	2.5	4.5	0.9924
SA12	Trachyte	6.82984	39.79814	4050	5.0	5.0	3.5	4.0	0.9992
SA13	Trachyte	6.82470	39.79431	4049	1.6	1.3	1.2	3.0	0.9991

### Lithic analyses

Terminal Pleistocene layers at all investigated archaeological sites yielded sufficient lithic material for analysis, including Dimtu (N = 446), Simbero (N = 308), and Webi Gestro (N = 474). Our study loosely follows a *chaîne opératoire* approach (Boëda et al. 1990; Inizan et al. 1999; Soressi and Geneste 2011), aiming at the direct comparison of all reconstructed stages of lithic transformation – from raw material acquisition and transport, through core preparation and reduction, blank production, tool manufacture and use, to final discard. The predominantly obsidian lithic material was studied through a combination of electron microprobe analysis, attribute-based technological analysis, and functional analysis, including use-wear and residue studies.

#### Geochemical analysis of obsidian

Electron microprobe analysis was carried out on 16 obsidian artifacts at the University of Utah using a Cameca SX-50 instrument, following established protocols in Eastern African provenance studies (Brown et al. 2013; Brown and Nash 2014; Negash et al. 2020). For each artifact, three points were analyzed on two fragments under conditions of 15 kV accelerating voltage, 25 nA beam current, and a 10–25 µm beam diameter. Oxygen was measured directly, with analytical totals providing both a quality check and an estimate of water content. Further details on methods, standards, and laboratory specifications are available in Kuehn et al. (2011). Apart from the 16 newly obtained results (Table S3), our comparisons included 61 previously published geological source samples (Negash et al. 2020). We excluded values from the Gira-Ale 2 volcano due to their exceptional composition. As an internal control, we added values of twelve artifacts from Fincha Habera (Fig. 1C) that have been securely identified as deriving from the Bale Mountains’ local Wasama obsidian outcrop (Ossendorf et al. 2019). As a first step, Al₂O₃/Fe₂O₃ ratios were used to separate northern Afar Rift sources (> 4.7) from MER sources (< 4.0), providing a preliminary assignment to large-scale landscape units and helping to flag outliers (Negash et al. 2020; Negash 2022). Because geochemical data are compositional (oxide percentages subject to the closure problem), statistical approaches requiring large group sizes – such as canonical discriminant analysis – are unsuitable. Instead, we applied a multistep workflow designed for individual samples and uneven group sizes, also to comply with the *provenance postulate* (see Frahm 2025): Principal component analysis (PCA) reduced dimensionality and highlighted the main patterns of variance, yielding an interpretable overview of sample–source relationships (Jansson et al. 2022; Tomczyk and Żabiński 2024). To complement this, we used non-metric multidimensional scaling (NMDS) with both Euclidian and Chord distance, which is well-suited for datasets with many small groups, as often encountered in provenance studies (Bialik et al. 2021; Vermeesch et al. 2023). Finally, Ward’s hierarchical clustering (WHC) was applied to minimize within-group variance and produce compact, well-separated clusters. In combination with PCA and NMDS, WHC enhances both dimensionality reduction and the identification of coherent compositional groupings (Disser et al. 2016; L’Héritier et al. 2020; Tomczyk and Żabiński 2024). All statistical analyses were carried out using the PAST v5.2.2 software (Hammer et al. 2001).

#### Technological analysis

Lithic technological analysis was carried out independently for each of the early AHP assemblages, following the recording protocol described in Ossendorf et al. (2019). In the interest of clarity and comparability, the following terminological and classificatory simplifications were applied, with no intention of proposing a new or universally applicable framework: First, we deliberately refrain from using the term *Later Stone Age* (LSA) due to (1) the lack of a consistent and practically applicable definition of the LSA (Leplongeon et al. 2023), and (2) the fact that most existing definitions are based on research in East Africa (Ambrose 1998; Diez-Martin et al. 2009; Tryon 2019; d’ Errico et al. 2020; Sahle 2020). Even the most recent attempt to distinguish Eastern African MSA from LSA assemblages – based on artifact type combinations and broadly defined technological criteria (Grove and Blinkhorn 2020) – includes only one LSA site (Aladi Springs) from the Horn. This is problematic, as distinct cultural trajectories have been noted for the Horn of Africa compared to other parts of Eastern Africa (Pleurdeau et al. 2014, 2023; Brandt et al. 2017; Tryon 2019; Shea 2020; Leplongeon et al. 2020a, 2020b, 2025). Second, we avoid problematic and inconsistently applied distinctions, such as *macrolithic* vs. *microlithic*, or *large backed pieces* vs. *small backed pieces* (the latter sometimes designated as *microliths*) which lack terminological consistency in the Horn of Africa (Leplongeon 2014). Instead, we provide metric data on cores, blanks, and selected tools. Third, the term *bladelet* is employed here in a strictly descriptive, morphological sense to denote small, elongated blanks (length ≥ 2 × width; width < 12 mm) derived from opportunistic core reduction of nodules, rather than from formal prismatic bladelet cores or pressure-flaking technologies. Although these products could alternatively be classified as *elongated blanks/flakes*, the term *bladelet* is retained because it more accurately reflects their regularized morphology in contrast to the otherwise predominantly opportunistic flake-based reduction strategies documented at the sites investigated here. We intentionally avoid alternative terms such as *microblade*, *small blade*, *laminar blank*, as well as arbitrary dimensional cut-offs for blades. This is especially appropriate given that none of the assemblages contain blanks with a length-to-width ratio of 2:1 and, simultaneously, a width exceeding 12 mm. Fourth, we understand *tools* as either *utilized* (macroscopic use wear), *retouched* (e.g., scrapers, notched pieces), or *backed* artifacts, reserving the term *shaped* exclusively for pieces that exhibit facial retouch. Fifth, *backed tools* are not subdivided into typological categories (e.g., *crescent*, *segment*, *backed blade(let)*, *micro-segment*) simply because (1) the lack of a unifying typological system in the literature, and (2) the morphological variability among the assemblages’ actual backed tools precluded consistent classification by all analysts. Similarly, we avoid the term *geometric* (microliths). The term *bipolar* is used exclusively for products of the bipolar anvil technique, and not for bidirectional core reduction strategies. Bipolar cores are recognized following de la Peña (2015) by features indicative of anvil-supported reduction, including platform notching or crushing at both ends, opposed removals along the full core length, step fractures, and splintered platforms. This identification method is consistent with approaches used in Eastern African assemblage studies (Eren et al. 2013; Shea 2013; Leplongeon 2014; Tryon and Faith 2016). Finally, *opportunistic reduction* is defined here as the flexible exploitation of available core surfaces with minimal preparation and no predetermined volumetric concept (after Andrefsky 2005), typically associated with multi-platform cores and irregular flake scarring. This strategy is well documented in Eastern African LSA bladelet-rich assemblages alongside formal microlithic production (Ambrose 2002; Barham & Mitchell 2008).

#### Use wear and residue analysis

The preliminary functional analysis was conducted to assess the preservation of residues and use-wear in the lithic assemblages. The study targeted samples of lithics selected by the excavators from Simbero (n = 41) and Webi Gestro (n = 10). Only the artifacts deriving from layer V at Simbero (n = 7) and layers IV-V at Webi Gestro (n = 5) that relate to the short terminal Pleistocene settlement phase (c. 15–14 ka) are considered here. The Simbero subsample comprised six backed pieces and one utilized bladelet. The Webi Gestro subsample consists exclusively of utilized bladelets (some fragmentary). Apart from a single chert artifact in the Webi Gestro sample, all the lithics are in obsidian. The lithic artifacts (n = 12) were examined with a stereomicroscope (Zeiss V12), a macroscope (V16) and an incident light microscope (Zeiss AxioImager) to record residues in situ. Residue analysis followed established protocols (Fullagar 2014; Langejans 2011; Cnuts and Rots 2018), focusing on smearing, association with use-wear, and distribution patterns. Potential functional residues were further analyzed with scanning electron microscopy (SEM) and energy-dispersive X-ray spectroscopy (EDX) (Borel et al. 2014; Pedergnana and Ollé 2018; Hayes et al. 2019; Hayes and Rots 2019) using a scanning electron microscope JEOL IT300 (EDX detector JEOL ex-230). Images and elemental spectra of the residues were acquired in situ on the tool surface in low vacuum (LV) mode (100 Pa) using the backscattered electron detector (BED) at 20.0 kV with a probe current (PC) of 60.0. Use-wear analysis proceeded in two stages: (1) low magnification (8–100 ×) under a stereomicroscope (Zeiss V12) to record functional wear and potential post-depositional damage, and (2) brief inspection with high magnification (100–200 ×) under an incident-light microscope (Zeiss AxioImager) to confirm preliminary observations and evaluate preservation. Use-wear features were imaged under low magnification and oblique lighting with a Zeiss Macro-Zoom Macroscope V16 and z-stacked in Helicon Focus software. High magnification images were captured with the Zeiss AxioImager, microscope camera AxioCam ICc 5 and software Zeiss AxioVision and stacked in the same software. No chemical cleaning necessary for a full high magnification analysis was carried out due to the presence of potential functional residues on the artifacts that were to be preserved for further analysis. This limited the observation possibilities for artifacts with persistent sediment remains and other surface deposits and highlights the preliminary nature of use-wear interpretations. Surface preservation was evaluated here on a preliminary basis through stereomicroscope and incident light microscope observation and categorized as good (little difficulty in analysis), moderate (taphonomic features partly obscuring wear), or poor (limited interpretability at high magnification) for the purposes of evaluating use-wear potential, without an attempt to characterize natural and anthropogenic processes responsible for the observed alterations. Tool use was inferred from wear patterns consisting of low-magnification features (edge rounding, scarring) and high-magnification evidence (linear traces, abrasion, polish) (Semenov 1964; Tringham et al. 1974; Hayden 1979; Odell and Odell-Vereecken 1980; Odell 1981; Keeley 1980; Vaughan 1985; Knutsson 1988; Hurcombe 1992; Juel Jensen 1994; Beyin 2010; Rots 2010; Brito-Abrante and Rodríguez-Rodríguez 2024). Besides use, various taphonomic processes can cause fracturing of the brittle edges of obsidian artefacts. Particularly trampling that can inflict significant damage on glassy materials (Knudson 1979) has been addressed in previous experimental studies. These works demonstrate that trampling removals generally have random distributions, although their tendency to propagate along existing ridges and convexities can sometimes cause them to cluster into pseudo-retouch (Flenniken and Haggarty 1979; Gifford-Gonzalez et al. 1985; Pryor 1988; McBrearty et al. 1998). Nevertheless, there is a consensus among authors that use and trampling scars are distinguishable by their distribution and attributes (Pryor 1988). We considered here scars that showed a patterned distribution and a clear association of microscopic wear (particularly systematically oriented linear features) as use-related (cf. Hurcombe 1992). Patterned scarring that could not be coupled with diagnostic microwear due to surface preservation issues or unclean tool surfaces are reported with caution. Linear features form readily on obsidian (Tringham et al. 1974) and are useful indicators of use action (Hurcombe 1992). They are sometimes detectable under low magnification (Fedje 1979; Beyin 2010) and can be further characterized with incident light and scanning electron microscopy (e.g. Fedje 1979; Hurcombe 1992; Brito-Abrante and Rodríguez-Rodríguez 2024). While certain tool use activities such as hide-scraping can produce microscopic linear features with highly variable directions (e.g. Pichon et al. 2025 fig. 10a-b), we focused the attention here only on clusters of linear features showing a shared orientation that combines logically with the direction and position of macroscopic damage, as linear features with random orientations occur on obsidian artefacts as a result of taphonomic processes (e.g. Hurcombe 1992 p. 80). Comparisons were also made between microscopic features observed on the supposed used edges and those occurring on inner surfaces and ridges of the artefacts to further rule out a taphonomic origin for the observed traces (Hurcombe 1992 p. 75). Due to the differences between investigators in the use of terminology for describing microscopic linear features on obsidian and other raw materials (see e.g. Hurcombe 1992; Knutsson 1988), we refrain here from using precise categories and refer to these traces simply as *linear features*. Projectile identification was based on the observation of wear patterning and attributes of individual features, acknowledging the complications involved in taking single fractures as evidence for projectile impact due to equifinality in damage formation through processes such as knapping, projectile use, and trampling (Fischer et al. 1984; Pargeter 2011; Rots and Plisson 2014; Coppe and Rots 2017; Taipale and Rots 2019). Only artifacts with several macroscopic breaks or scars indicative of significant compression involved in fracturing, forming a consistent pattern, and associated with linear features initiating from the terminations of the fractures were ranked here as certain projectiles. Artifacts displaying more limited sets of features (e.g. only macrofractures or ambiguous macrofractures associated with microscopic linear features) and no indications of other kinds of use are reported as possible projectiles. Previously published experimental results on backed obsidian armatures (Goldstein & Shaffer 2017; see also Yamaoka 2017 for different morphology) informed the interpretations proposed here. No tailored experiments were carried out at this stage with Bale Mountains raw materials. Although previous experimental work on obsidian demonstrates that macroscopic use damage formation follows same general principles as those documented for other rocks, such as the higher incidence of abrupt scar terminations in working hard contact materials (see e.g. Tringham et al. 1974; Broadbent and Knutsson 1975; Beyin 2010; Brito-Abrante and Rodríguez-Rodríguez 2024), and microwear studies demonstrate links between combinations of microscopic features and worked material properties (Hurcombe 1992; Brito-Abrante and Rodríguez-Rodríguez 2024), we refrain here from inferring the properties of worked materials from scar attributes and microwear features on obsidian in the absence of a tailored physical reference collection. This acknowledges the overlap between linear feature characteristics across different worked materials and the added variability introduced by e.g. grit (Hurcombe 1992), which make use-wear diagnostics relying only on published images unfeasible. In contrast, chert use-wear interpretation relied here on the extensive reference collection TRAIL housed at TraceoLab (Rots 2021).

## Results

### Deglaciation history of the Sanetti Plateau

**Fig. 4:**
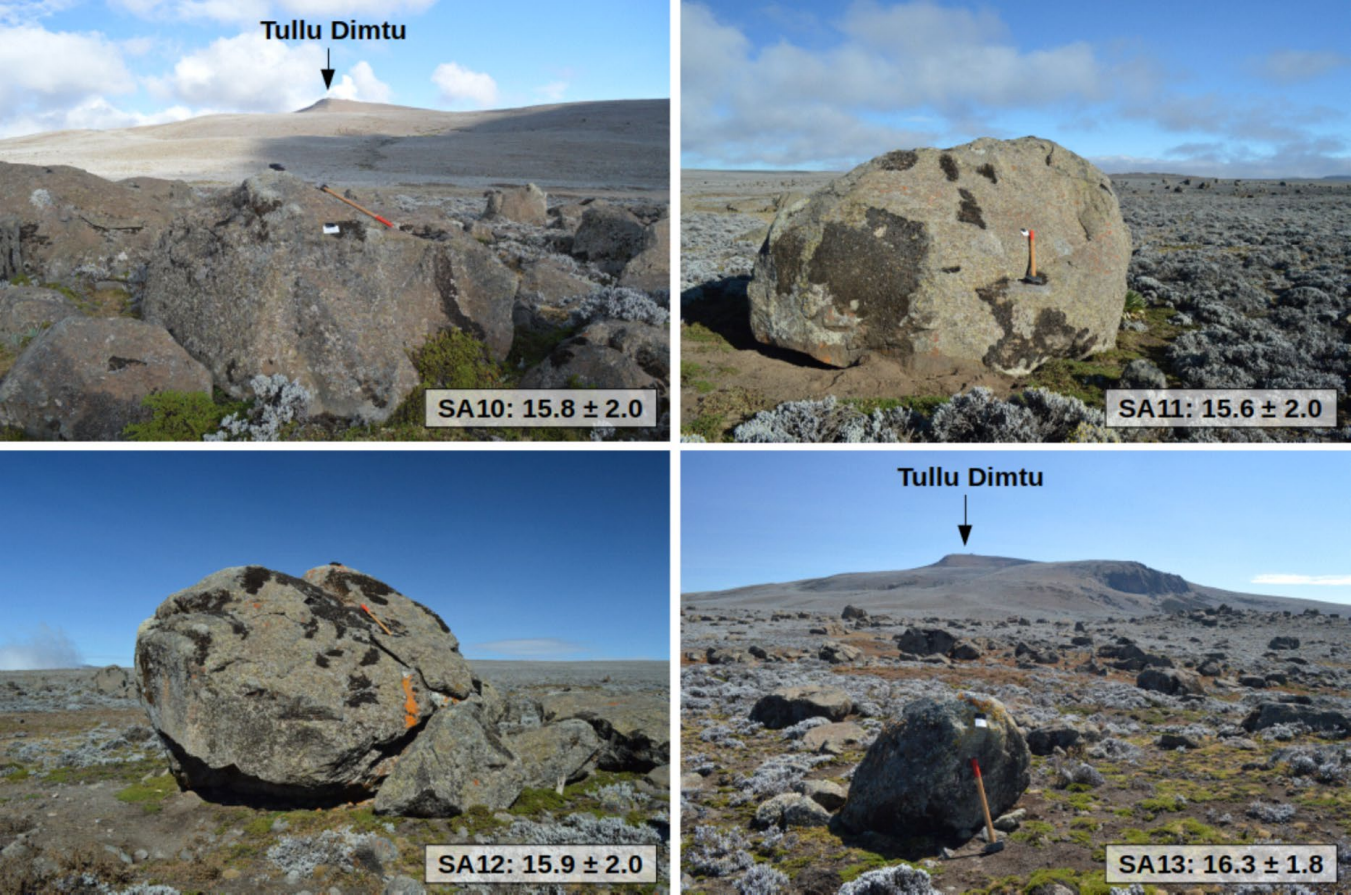
Photos of the four dated boulders (SA10-13). They belong to the inner Big Boulder Moraine west of Tullu Dimtu on the Sanetti Plateau (Fig. 1C)

While the evolution of the ice cap and its northern outlet glaciers after the *local* LGM (42–28 ka) remains partly unclear (see Sect. "The study area: Bale Mountains (south-central Ethiopia)".), published exposure ages from well-preserved moraine sequences in the northern valleys, together with radiocarbon ages from lacustrine sediments, indicate that deglaciation in the Bale Mountains set in around 18–17 ka (Fig. 1C). The four newly dated boulders from the plateau (SA10-SA13; Fig. 4) yield consistent ages of 15.8 ± 2.0 ka, 15.6 ± 2.0 ka, 15.9 ± 2.0 ka, and 16.3 ± 1.8 ka (Table 4). These results allow, for the first time, a precise and unambiguous determination of the landform age of the inner Big Boulder Moraine (15.9 ± 0.3 ka), which marks the onset of the final recession stage of the central ice cap during deglaciation of the Sanetti Plateau. The striking synchronicity between the retreat from the innermost moraines in the northern valleys at 16.0 ± 1.0 ka, the onset of sedimentation at 15.9 ka cal. BP in Lake Garba Guracha (Fig. 1C), and the disintegration of the central ice cap after 15.9 ± 0.3 ka provides strong evidence that the Bale Mountains became ice-free (except for possibly small cirque glaciers or perennial snowfields at the highest peaks that cannot be ruled out) by the time of arrival of early AHP settlers. By 15 ka, hunter-gatherers inhabited an extensive, open Afroalpine landscape dominated by shrubby and herbaceous vegetation (Fig. 1B).

**Table 4:** Cosmogenic ^36^Cl data and surface exposure ages of the four rock samples from the Sanetti Plateau

Rock sample	Rock dissolved (g)	^35^Cl spike (mg)	Cl (ppm)	^36^Cl (10^5^ At g⁻^1^)	Exposure age (ka)
SA10	30.0465	3.5243	212.93 ± 1.44	16.46 ± 0.56	15.8 ± 2.0
SA11	30.4160	3.5231	200.79 ± 1.59	16.03 ± 0.54	15.6 ± 2.0
SA12	30.0424	3.5225	176.25 ± 2.10	15.08 ± 0.53	15.9 ± 2.0
SA13	30.0175	3.5298	137.19 ± 1.94	13.67 ± 0.48	16.3 ± 1.8

### Provenance of obsidian artifacts

**Fig. 5:**
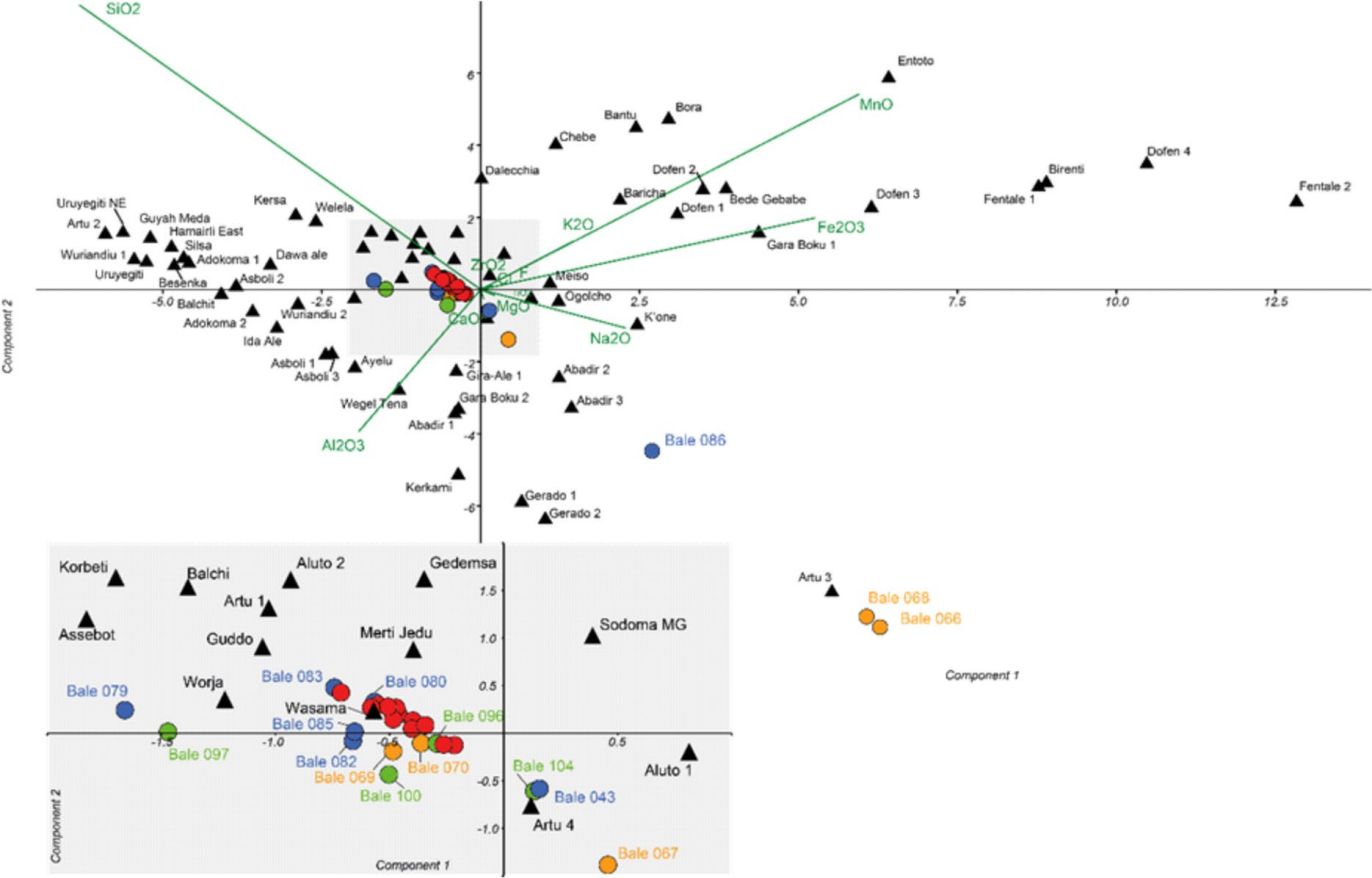
Biplot of the principal component analysis (PC1 vs. PC2) of the geochemical dataset. The respective loading vectors are represented by green lines. Obsidian sources/volcanoes are shown as triangles and archaeological samples as circles. Colors indicate sample provenance according to the archaeological site (Dimtu = orange, Simbero = green, Webi Gestro = blue, Fincha Habera = red). The central cluster (shaded area) is enlarged below. See Table S3 and Fig. 3 for archaeological sample provenance

**Fig. 6:**
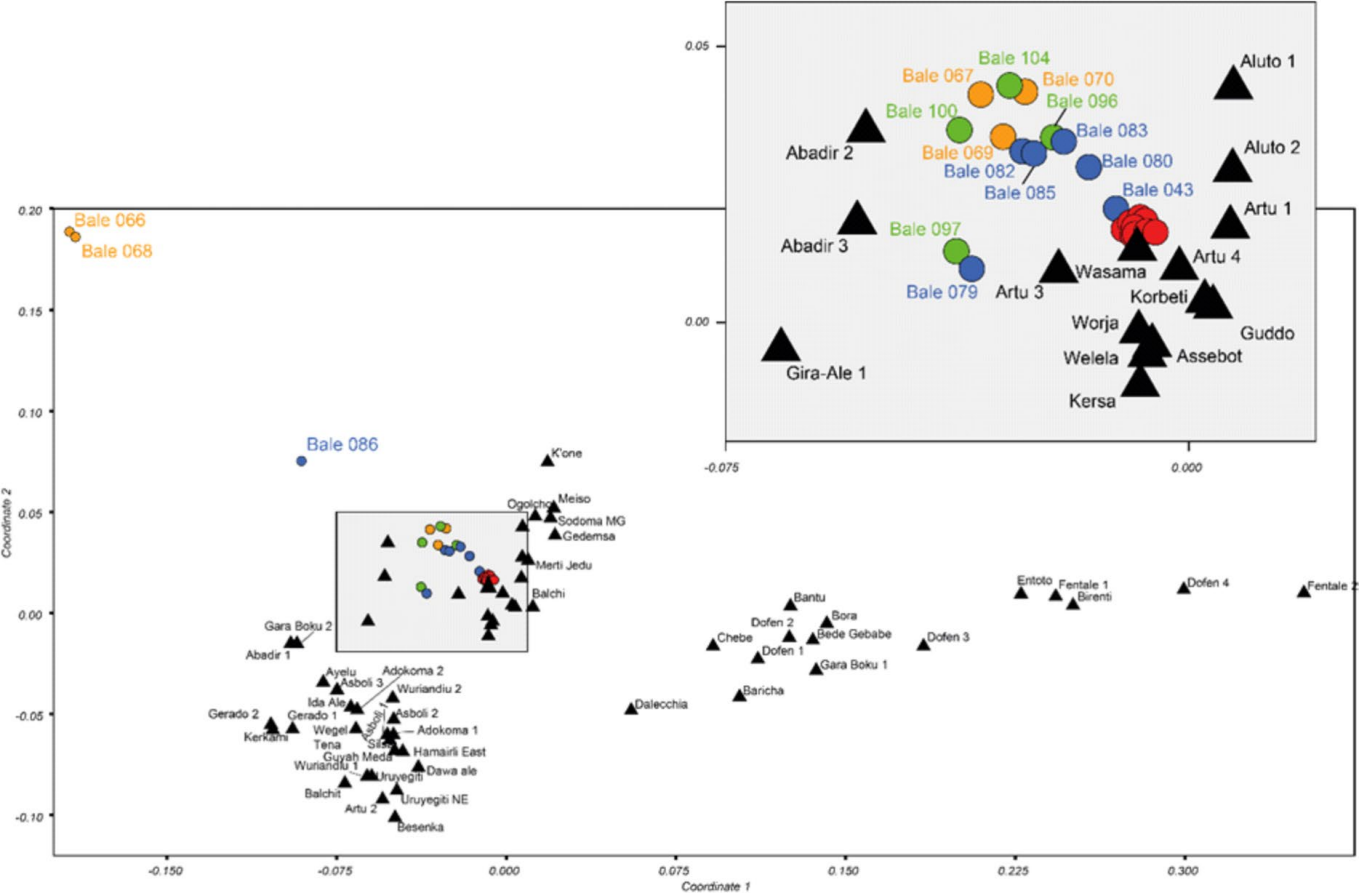
NMDS ordination of obsidian samples based on geochemical composition. The central cluster (shaded) is enlarged in the upper part of the figure. Archaeological sites are shown as circles, and obsidian sources as triangles. Samples from archaeological sites include Dimtu (orange), Simbero (green), Webi Gestro (blue), and Fincha Habera (red). See Table S3 and Fig. 3 for archaeological sample provenance

**Fig. 7:**
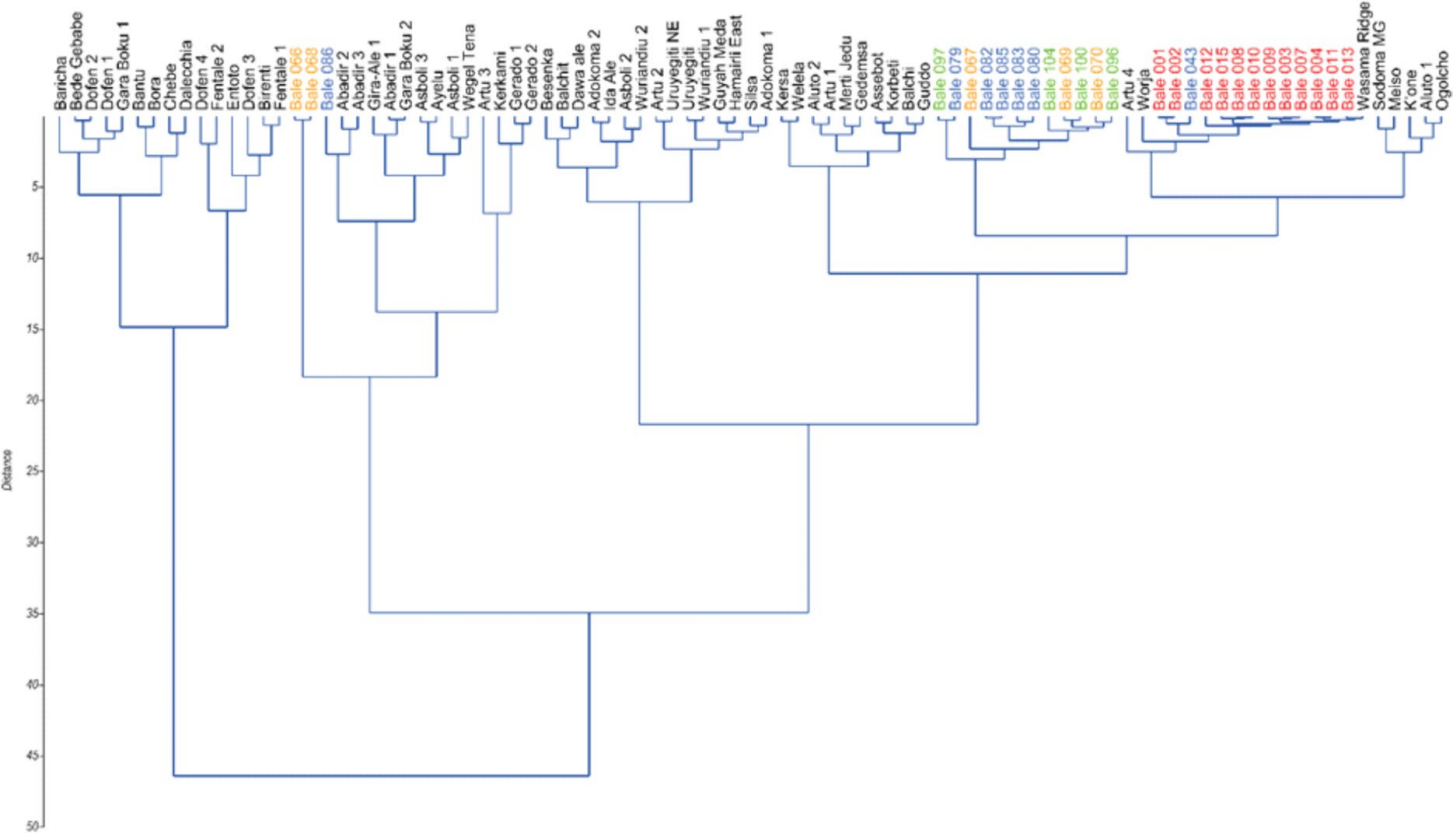
Hierarchical cluster analysis (Ward’s method, Chord distance) of obsidian samples and geological reference sources. Sources are labeled in black; archaeological samples are shown in orange (Dimtu), green (Simbero), blue (Webi Gestro), and red (Fincha Habera). See Table S3 and Fig. 3 for archaeological sample provenance

The newly obtained electron microprobe analysis results (Table S3) show that all samples have Al₂O₃/Fe₂O₃ ratios below 4, consistent with sources in the MER south of 9.5°N (Negash et al. 2020). A provenance from the northern Ethiopian Afar Rift can therefore be excluded for all the samples. Three samples (Bale 066, 068, 086) are clearly distinguished by their markedly low SiO₂ contents, coupled with elevated TiO₂, Al₂O₃, Fe₂O₃, Na₂O, and K₂O levels. The principal component analysis (PCA) of the geochemical dataset shows that the first two PCs explain more than 80% of the total variance (Table S4), which allows for a meaningful two-dimensional representation of the data (Fig. 5). Including the third component increases the explained variance to 92.3% (Table S5). PC1 contrasts high SiO₂ and MgO contents (negative loadings) with elevated Fe₂O₃ and MnO (positive loadings), representing the dominant axis of variation between silica-rich compositions and Fe–Mn-enriched glasses. PC2 is driven by a strong positive loading on SiO₂ and a negative loading on Al₂O₃, distinguishing highly silica-rich obsidians from those with relatively higher alumina contributions, potentially linked to feldspar fractionation. PC3 separates Al₂O₃ (positive loadings) from Fe₂O₃ and Na₂O (negative loadings), capturing subtler differences between alumina-enriched and Fe–Na-bearing trends. Archaeological samples plot predominantly along the SiO₂ vector, reflecting a monotonic silica gradient that explains most of the variance captured by PC1 and PC2. Elements oriented opposite to SiO₂, such as Al₂O₃, covary inversely, consistent with known compositional trends in obsidian glasses. Most archaeological samples cluster near Wasama Ridge in the Bale Mountains. However, the older MSA Fincha Habera artifact samples (red circles) appear much more homogeneous in composition than the AHP samples (orange, green, and blue circles). The latter are sometimes closer related to sources along the MER volcanoes, particularly Artu 4 (samples Bale 043 and 104), and to a lesser extent Guddo, Assebot and Worja (Bale 067, 079, and 097). Clear outliers with distinctly low SiO₂ contents include Bale 066, 068, and 086, the former two being almost identical in composition (Fig. 5).In the second step, instead of relying solely on linear distances, we applied non-metric multidimensional scaling (NMDS). NMDS projects the distances or similarities between samples from multidimensional space into a two-dimensional representation while preserving their relationships as accurately as possible. The resulting configuration shows an excellent fit to the distance matrix (stress = 0.064), providing a robust low-dimensional view of the geochemical variation among the obsidian samples. The Shepard plot (Fig. S1) shows a strong correspondence between the original dissimilarity ranks and the distances in the NMDS configuration, indicating a good model fit. The NMDS plot (Fig. 6) confirms the previously identified outliers (Bale 066, 068, 086), which remain well separated from all known obsidian sources in Ethiopia. Conversely, the closest match is again observed between Wasama and the Fincha Habera MSA samples (red circles in Fig. 6). The samples dating to the early AHP (circles in orange, green, and blue) form a looser cluster around Wasama, with some showing their shortest distances to the Artu 3/4 and Abadir 2/3 volcanoes. This pattern contrasts with the relationships identified by the PCA. Interestingly, NMDS also groups the known obsidian outcrops into three broader clusters. The first, and densest, cluster comprises sources from northern Ethiopia, especially the Afar Rift, together with a few sites from the northern Ethiopian Plateau. As already mentioned, most of these sources are known to also differ in their Al₂O₃/Fe₂O₃ ratios (Negash et al. 2020). The second cluster lies closest to the current archaeological samples investigated here and consists primarily, though not exclusively, of volcanoes from the central areas of the MER. The third cluster is more dispersed and is formed mainly by volcanoes from the northern sector of the MER (Negash et al. 2021). This arrangement suggests that, despite its overall complexity, the NMDS plot does retain a weak geographical signal. Finally, Ward’s hierarchical clustering (WHC) grouped the samples according to similarities in geochemical composition (Fig. 7). Hierarchical clustering using both Euclidean and Chord distance produced nearly identical dendrograms, indicating that the overall grouping of samples is robust to the choice of distance metric. The cophenetic correlation coefficient of 0.897 indicates a very good fit between the hierarchical clustering and the original distance matrix. The analysis supports the major groupings already identified by NMDS, although the finer branching patterns within clusters vary with this method and should not be overinterpreted. Importantly, cluster analysis confirms several key results from PCA and NMDS. The shortest distances occur between the Bale Mountains’ Wasama outcrop and the MSA samples from Fincha Habera, with Artu 4 and Worja identified as additional sources. Notably, the latter also includes the only AHP sample considered here (Bale 043 from Webi Gestro), a result consistent with NMDS but not with PCA. By contrast, most of the remaining AHP samples cluster together without a nearby geological source, echoing patterns observed in both PCA and NMDS. Their geochemical composition is even less similar to the local Wasama obsidian compared to e.g., Worja or Artu 4. The extreme outliers Bale 066 and 068 are again clearly isolated, while Bale 086 shows strong proximity to Abadir 2 and Abadir 3, consistent with the other methods’ results.

**Fig. 8:**
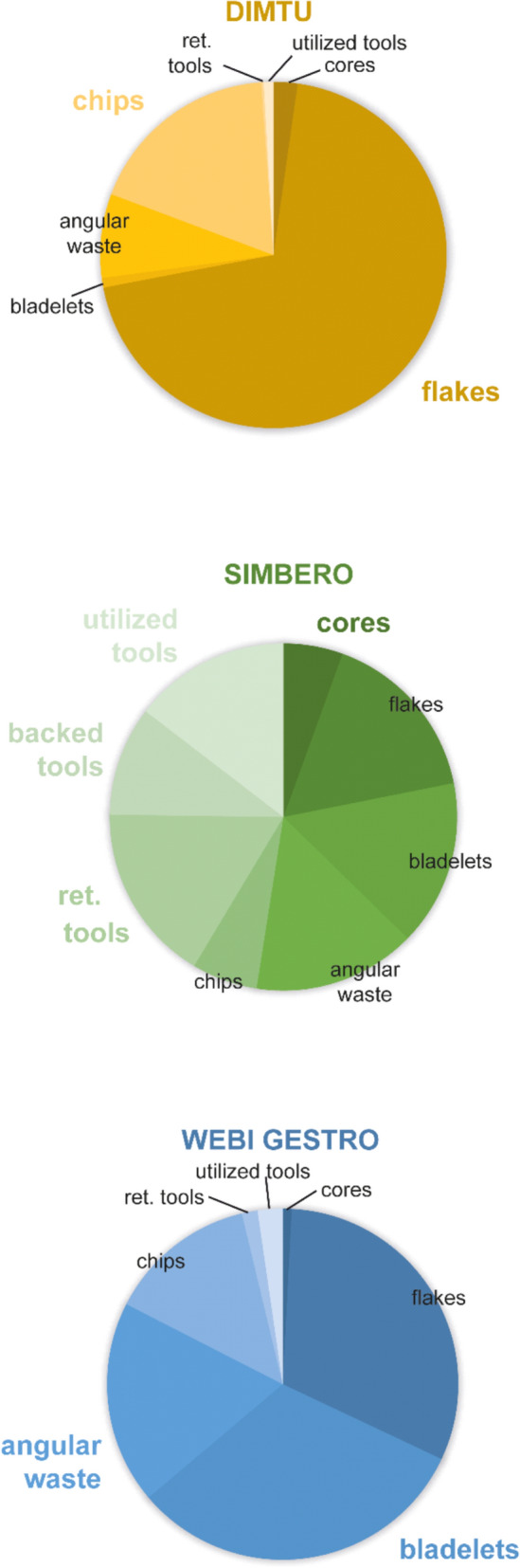
Compositional data of the three lithic assemblages. Percentage distribution of the main artifact categories in the assemblages of Dimtu (N = 446, orange), Simbero (N = 343, green), and Webi Gestro (N = 481, blue). Colored labels highlight the category with the largest share in direct comparison between assemblages

**Fig. 9:**
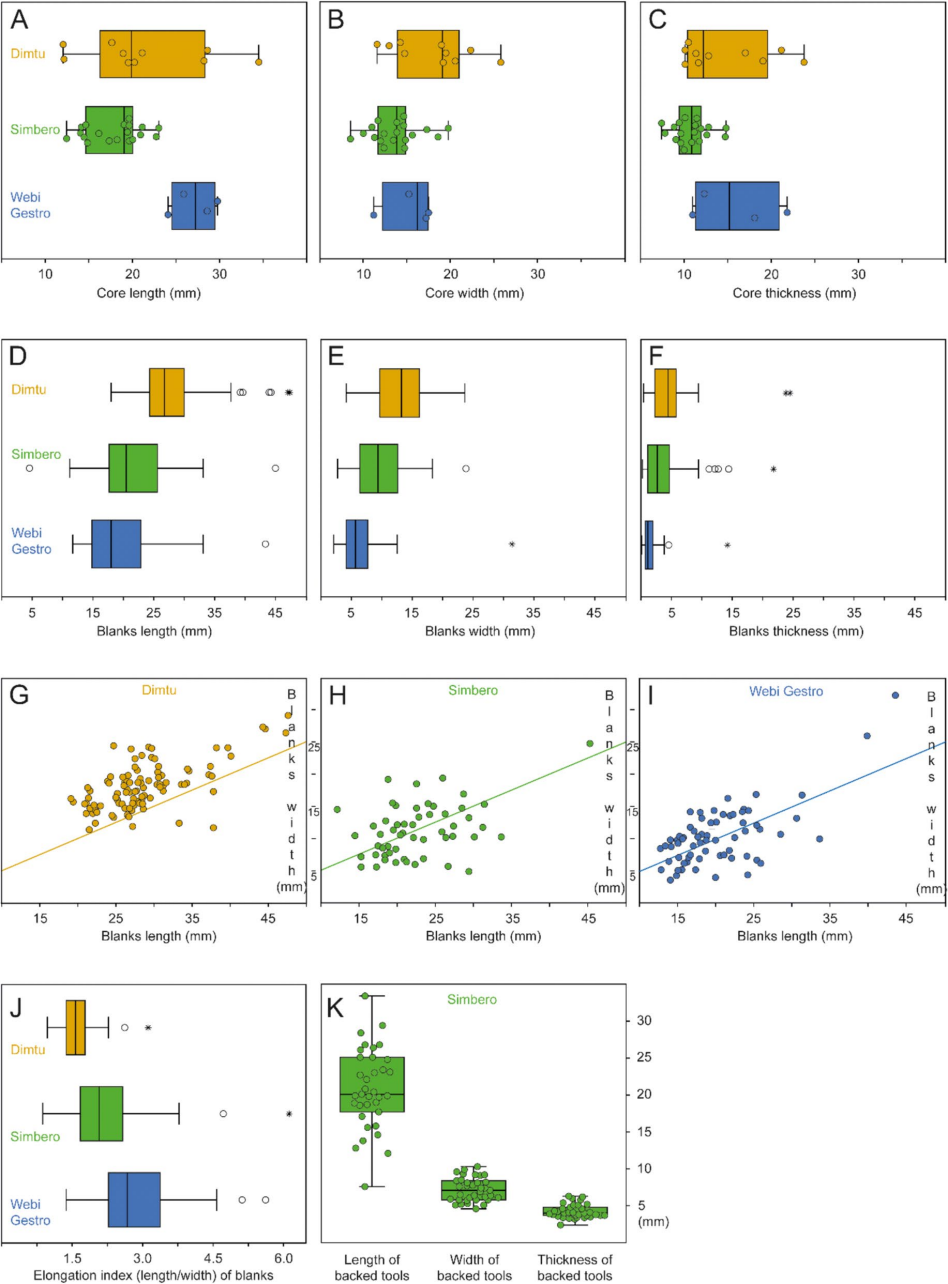
Summary of metrics from the three early AHP lithic assemblages. **A–C** Boxplots of core dimensions: length (mm) (A), width (B), and thickness (C) at Dimtu (N = 10, orange), Simbero (N = 19, green), and Webi Gestro (N = 4, blue). **D–F** Boxplots of completely preserved blanks: length (mm) (D), width (E), and thickness (F) at Dimtu (N = 69), Simbero (N = 55), and Webi Gestro (N = 100). Simple outliers are shown as open circles, extreme outliers as stars. **G–I** Scatterplots of blank dimensions: length (x-axis) vs. width (y-axis) (mm) at Dimtu (G, 2:1 ratio line included), Simbero (H), and Webi Gestro (I). **J** Elongation index (length/width) of blanks at all sites. **K** Measurements of backed tools (length, width, thickness in mm) from Simbero (N = 35). In the boxplots, open (unfilled) circles denote outliers and stars denote extreme outliers

**Fig. 10:**
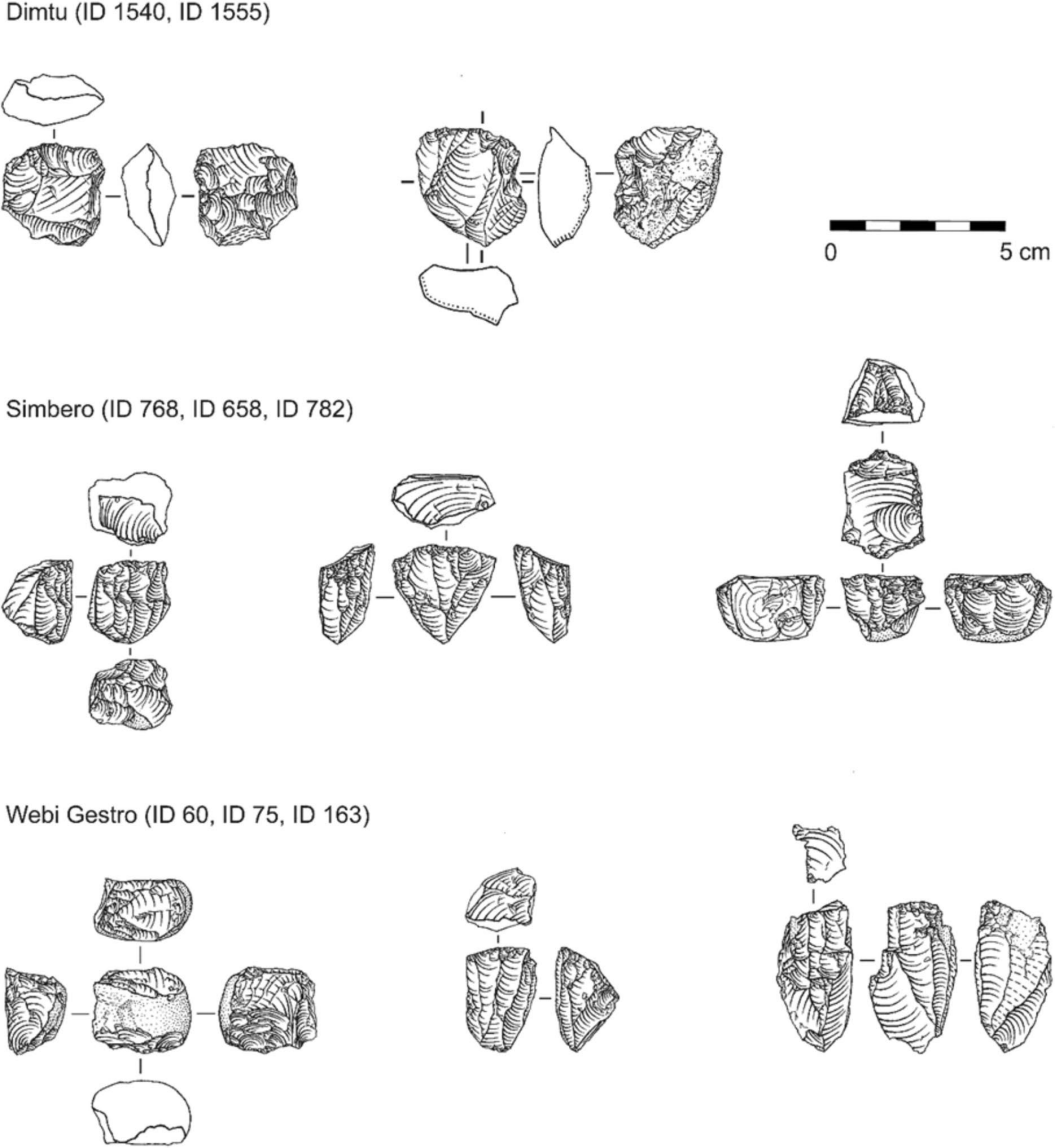
Selected typical obsidian cores from the three investigated sites. Artifact IDs are indicated from left to right. **Dimtu**: Core-on-flake (ID 1540) reduced using multiple encircling platforms; negatives indicate opportunistic, multidirectional exploitation. Irregular core (ID 1555) reduced from a single cortical platform, showing predominantly unidirectional negative organization. **Simbero**: Prismatic core (ID 768) reduced using opposed double platforms; parallel laminar negatives and evidence of platform rejuvenation indicate a prepared striking surface. Pyramidal core (ID 658) with opposed platform organization; convergent laminar negatives reflect maintenance through debordant removals. Prismatic core (ID 782) reduced from a single platform; parallel laminar negatives indicate intensive terminal exploitation, including bipolar reduction. **Webi Gestro**: Pebble core (ID 60) reduced using multiple encircling platforms; mixed flake and bladelet negatives indicate core rotation during exploitation. Pyramidal bladelet cores reduced from a single platform (ID 75, ID 60) with platform preparation; with convergent and parallel laminar negatives indicating unidirectional reduction sequences. Artifact IDs indicated from left to right. Short adjacent dashes indicate fracture surfaces (ID 1555), whereas dots/dotted lines denote the cortex (ID 60, ID 1555). All drawings by Ingrid Koch. Scale bar = 5 cm

**Fig. 11:**
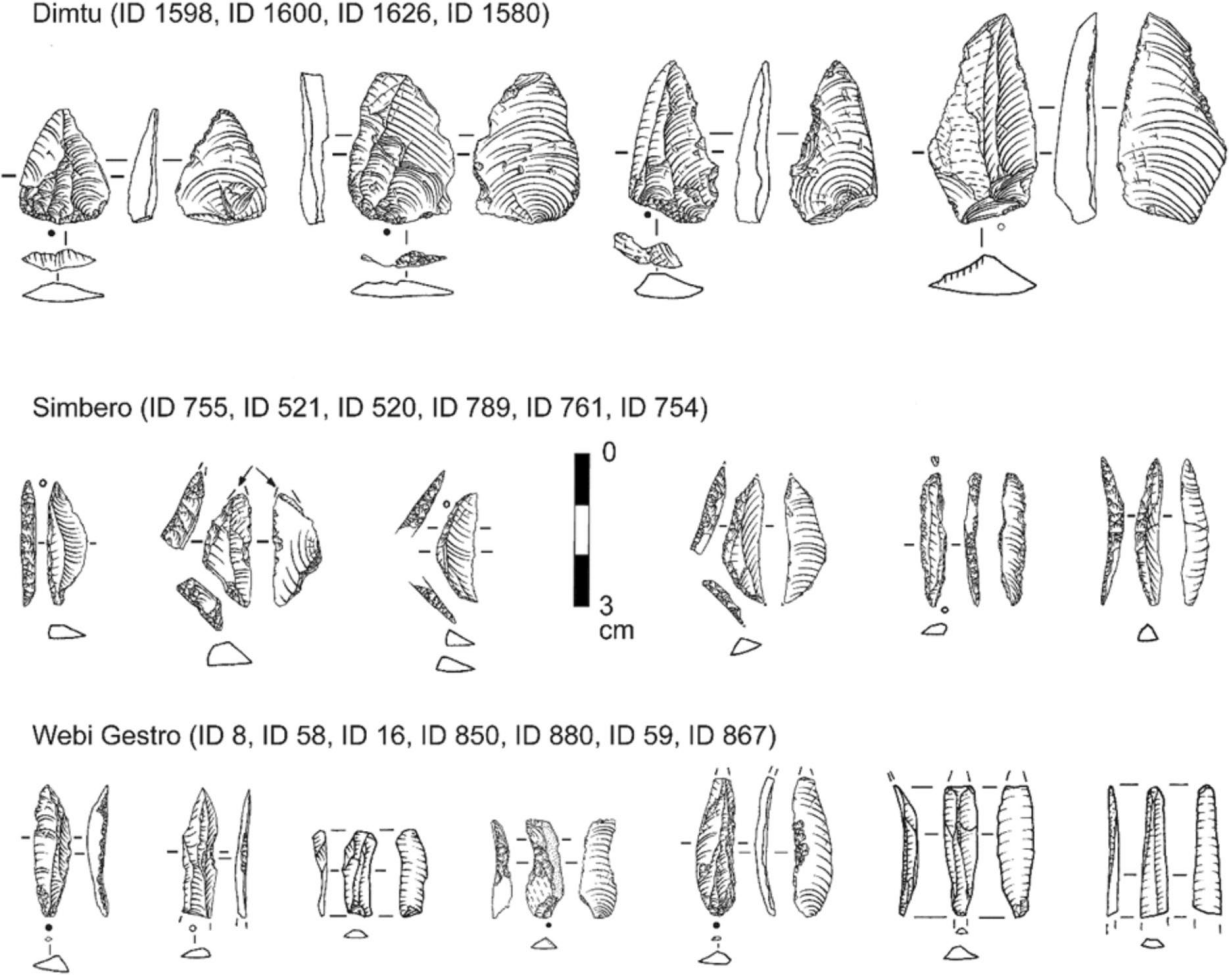
Selected typical obsidian blanks and tools from the three investigated sites. Top row: unretouched pointed flakes from Dimtu. Middle row: various backed tools from Simbero. Bottom row: variously retouched (mainly notched) and unretouched bladelets from Webi Gestro. Artifact IDs indicated from left to right. Circle positions indicate percussion direction; filled circles show preserved striking platform remnants, while open circles indicate no striking platform is preserved. Drawings by Ingrid Koch. Scale bar = 3 cm

### Lithic transformation stages

#### Raw material acquisition

**Fig. 12:**
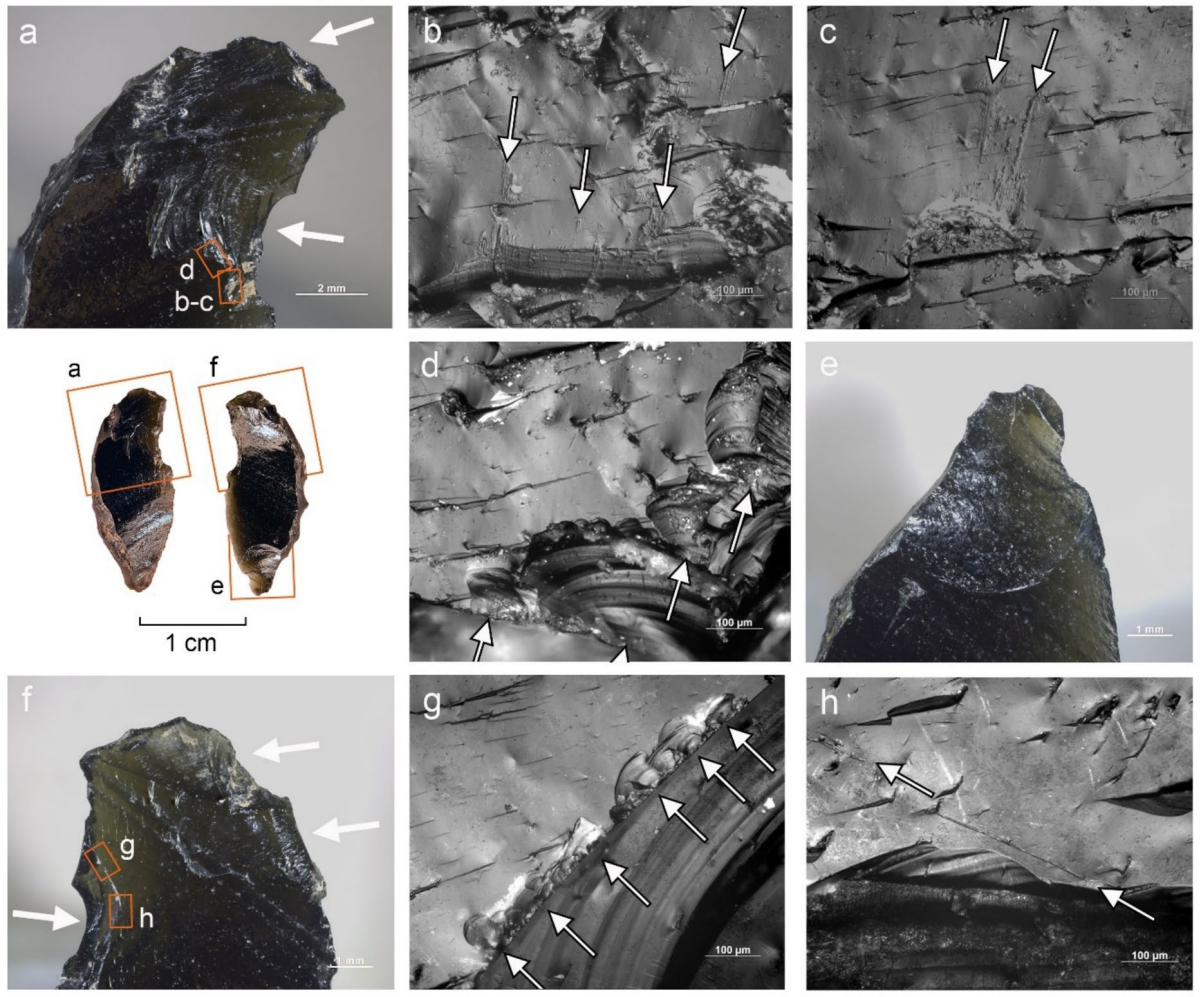
Evidence for projectile use on backed piece ID 537 from Simbero. **A** Invasive scars with abrupt, sometimes fissured terminations, initiated on the cutting edge and the backed distal extremity. **B**-**C** Microscopic linear features (arrows) initiated from the terminations of the scars on the cutting edge shown in A. **D** Secondary scars (arrows) associated with the macroscopic damage shown in A. **E** Overlapping removals at the proximal extremity, the largest of which could be production-related. **F** Invasive damage initiated on the cutting edge and the back on the ventral aspect of the distal end. **G** Small secondary scars with an oblique orientation associated with the perpendicularly oriented scars on the cutting edge shown in F. **H** Obliquely oriented linear features associated with the damage shown in F

**Fig. 13:**
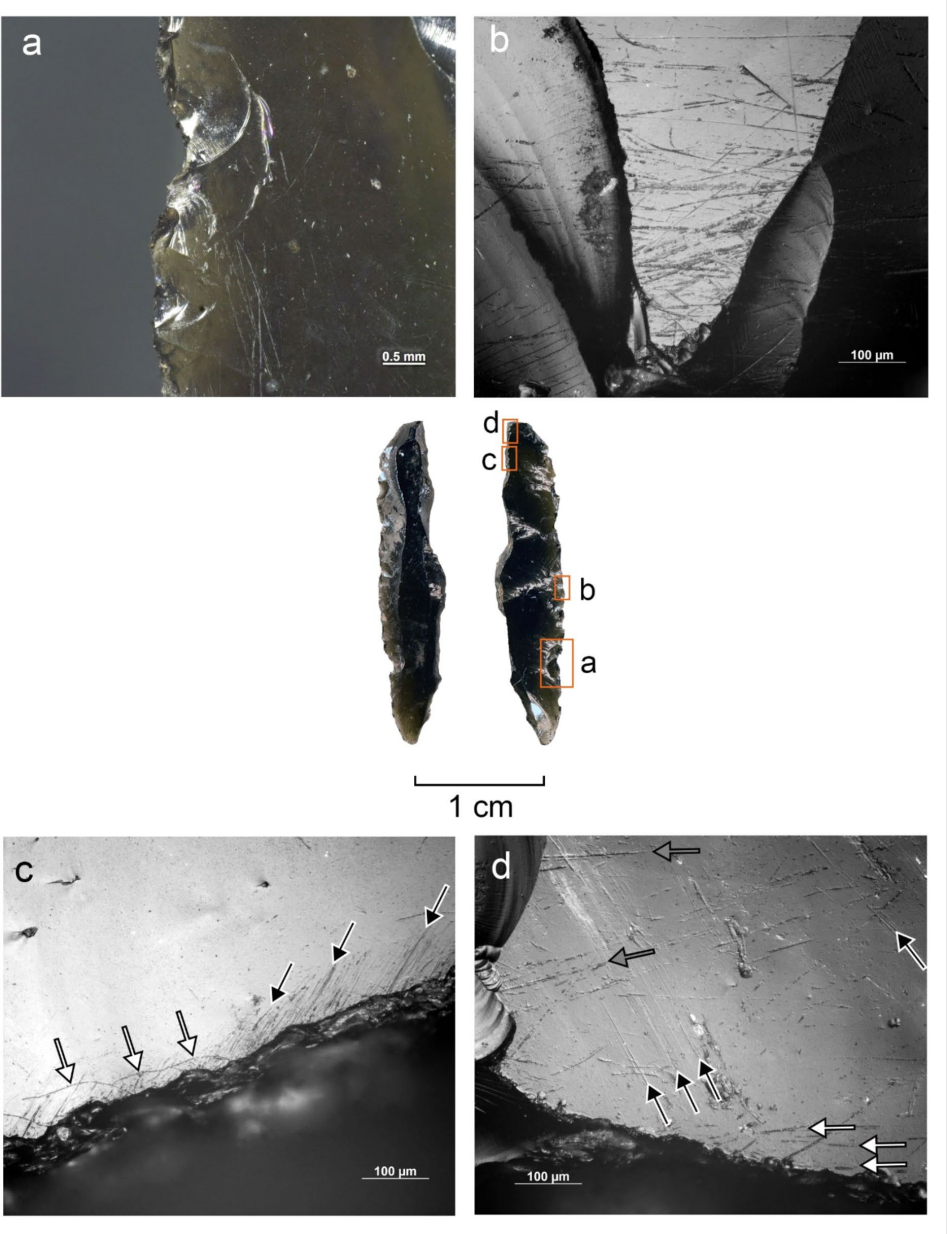
Use-wear on obsidian artifact ID 761 (Simbero). **A** Edge damage and striations on the ventral left edge (40 × , scale bar = 0.5 mm).** B** Linear features indicating longitudinal tool motion (200 × , scale bar = 100 μm). **C** Incipient cracks (white arrows) and oblique linear features (black arrows) from retouching on the ventral aspect of the backed edge. **D** Linear features with multiple orientations at the distal extremity. Black arrows mark probable retouch traces. Other features may derive from use (grey arrows) and hafting (white arrows; for somewhat comparable features, see Kononenko et al. 2015: fig. S5e) but taphonomic origin remains to be ruled out

**Fig. 14:**
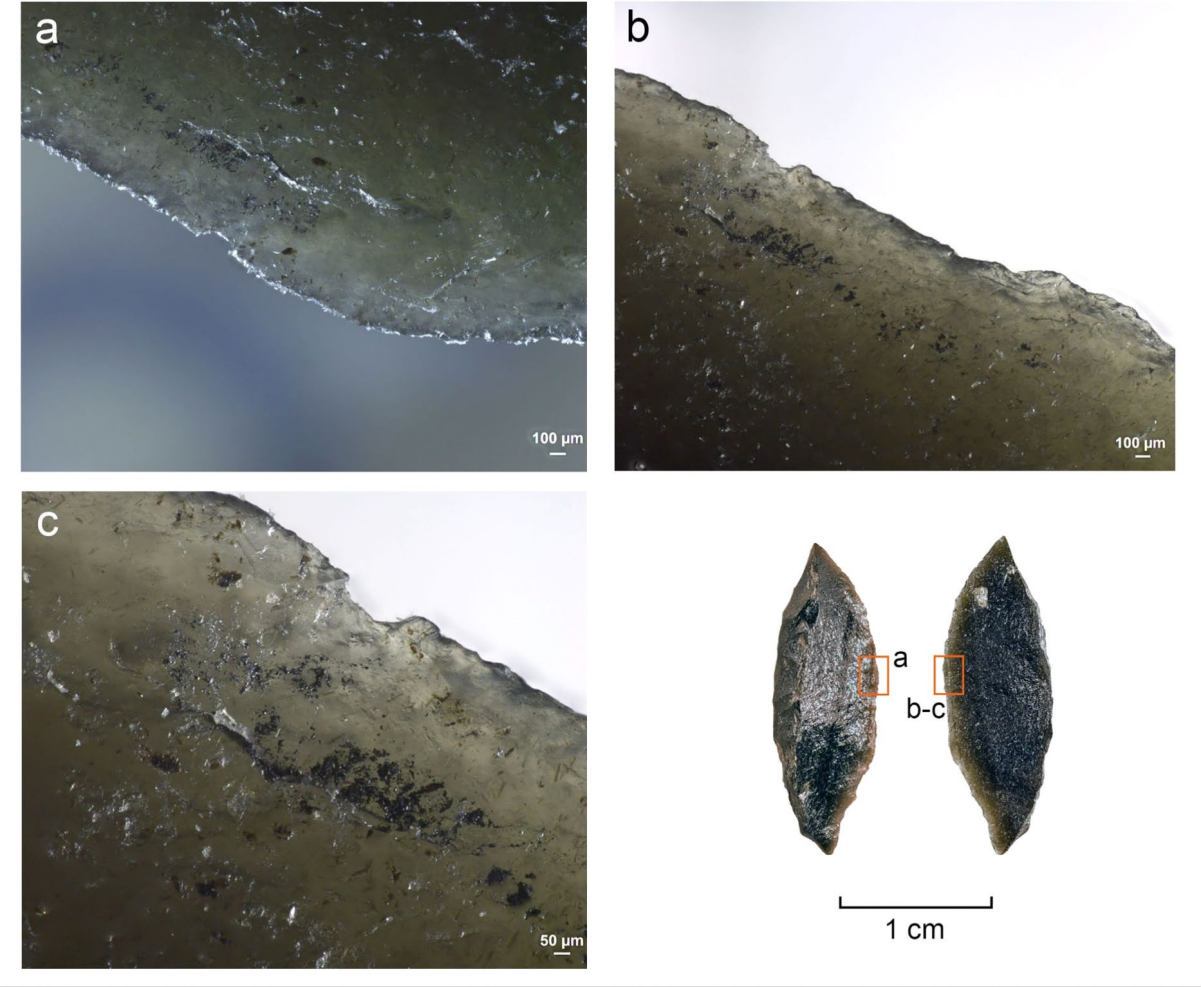
Possible use residue on backed piece ID 538 from Simbero.** A** Linear deposit of charred residue on the dorsal medial right edge. **B** Similar deposit on the dorsal aspect. **C** Detail of deposit shown in B

**Fig. 15:**
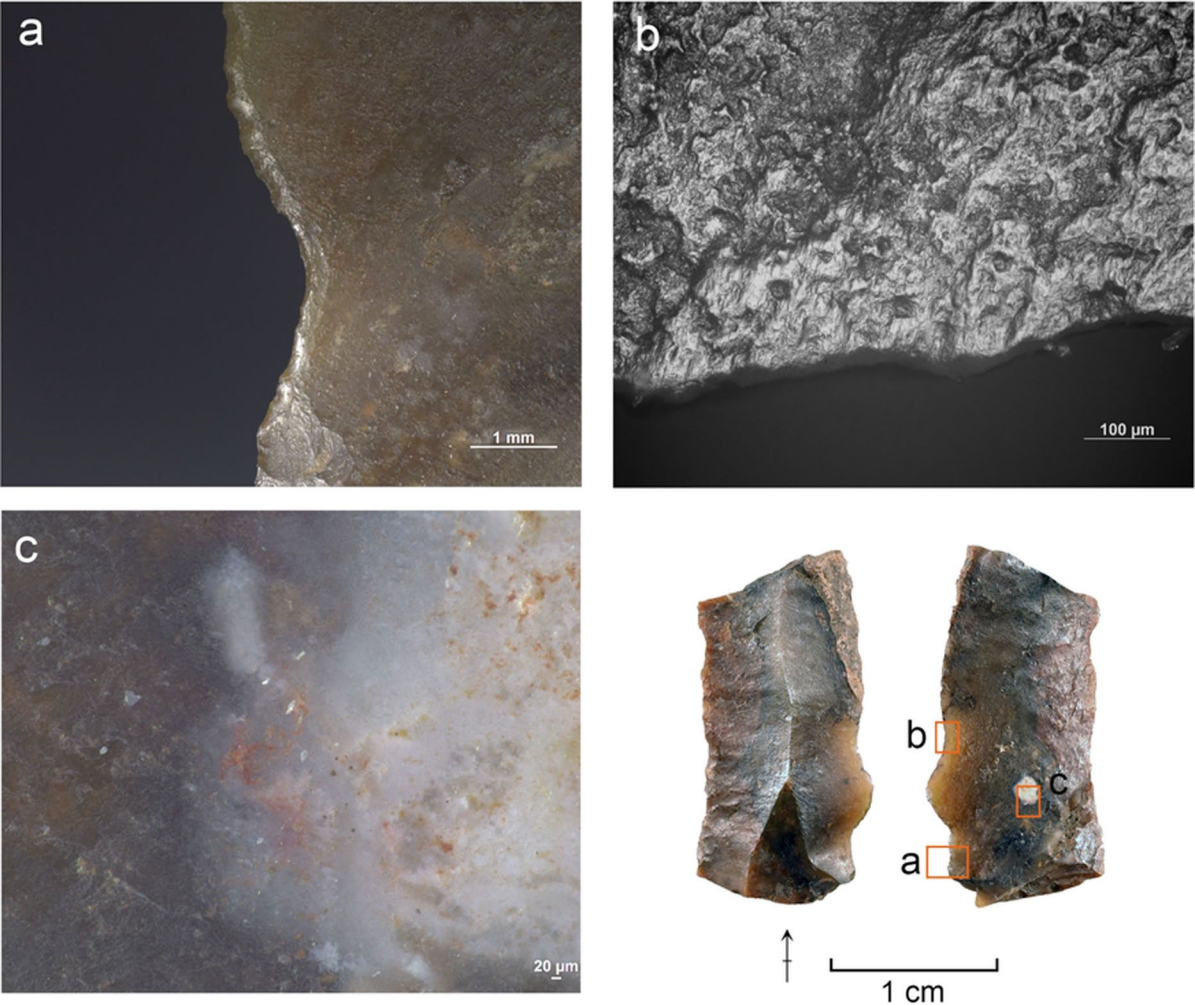
Use-wear and iron oxide residue on fragmentary bladelet ID 534 from Webi Gestro.** A** Bending-initiated scarring in the proximal part of the active edge. **B** Striated polish possibly from hide-working in the medial part of the active edge. **C** Iron oxide residue without informative distribution, potentially from the burial environment

**Fig. 16:**
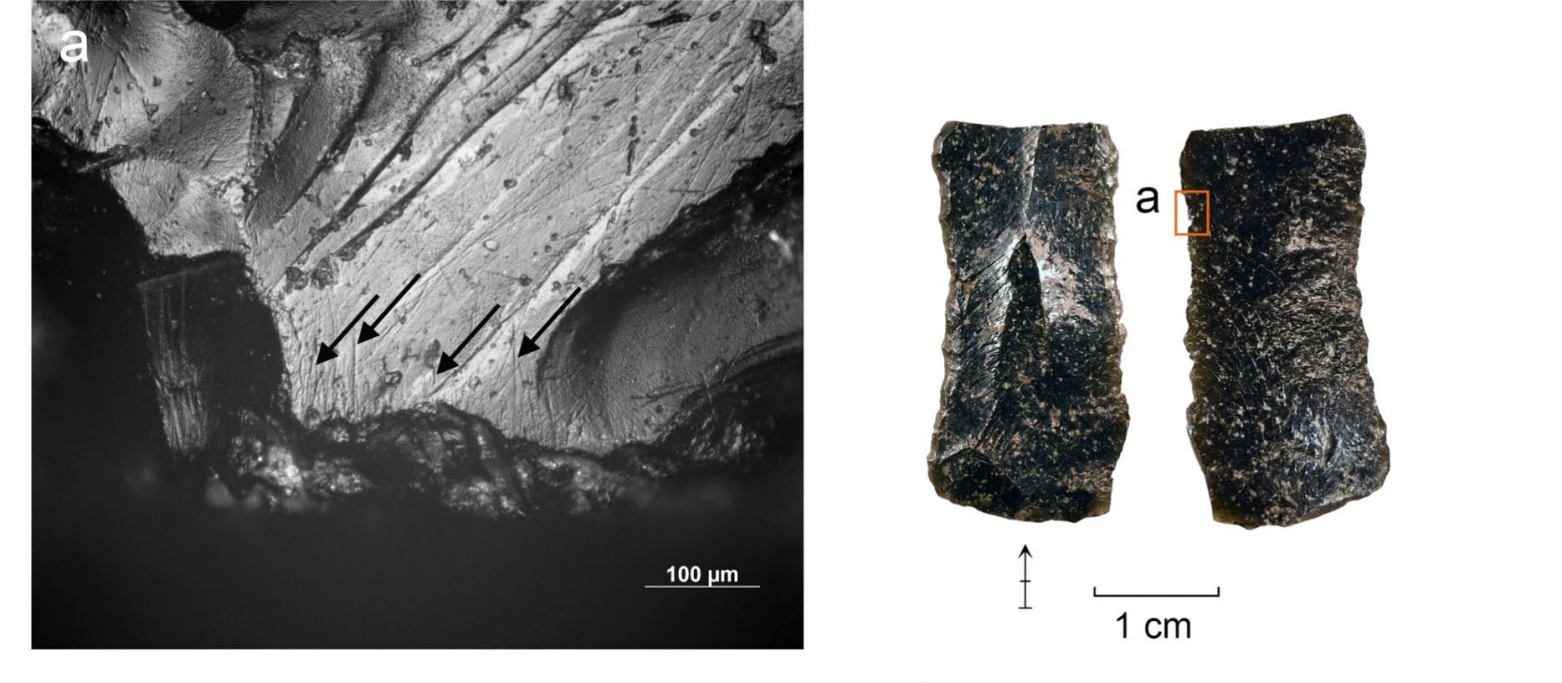
Microscopic linear features (black arrows) from transverse use on fragmentary retouched bladelet ID 535 from Webi Gestro

Obsidian raw material (see also Figs. 12–14,16), a naturally occurring volcanic glass known for its homogeneous vitreous texture and conchoidal fracture that produces exceptionally sharp edges (Heide and Heide 2011), dominates at all sites. Its provenance is not visually identifiable based on macro- or microscopic visible differences in texture. However, secondary sources, such as those observed in the Wasama and Mararo valleys (Fig. 1C), have also produced water-transported material in the form of cortical, rounded nodules. The recurrent occurrence of such small, only initially flaked nodules (less than 5 cm in size) found at Dimtu (N = 7) and Webi Gestro (N = 13), and the presence of similar pieces at Fincha Habera (Ossendorf et al. 2019, 2023), hint to local sources. Dimtu shows a notably higher proportion of non-obsidian artifacts (39.5%, Table 5), with > 90% of these being local chert from the Kore plain, located at a maximum distance of 12**–**20 km to the sites discussed here (Fig. 1C). Other materials, including chert varieties (see Fig. 15), chalcedony, basalt, quartz, and rhyolite, are found in small quantities, and their sources remain unidentified. In contrast, both Simbero (18.2%) and Webi Gestro (2.7%) show lower proportions of non-obsidian artifacts, with Kore chert again making up the largest share of non-obsidian materials, especially at Webi Gestro. Notably, cortical remains were almost entirely absent from the Simbero core assemblage despite it containing the largest collection of cores in terms of numbers and frequency (Table 5). At Dimtu and Webi Gestro, at least half of the cores exhibit cortical remains, with substantial cortical percentual coverage (Dimtu: > 30%, Webi Gestro: > 20%).

**Table 5:** Percentage distributions of assemblage composition and selected artifact characteristics across the three archaeological sites

Category	Subcategory	Dimtu	Simbero	Webi Gestro
Assemblage composition	Proportion cores	2.2	5.5	0.8
	Proportion blanks	70.6	31.8	63.0
	Proportion flakes	69.7	16.3	31.2
	Proportion bladelets	0.9	15.5	31.8
	Proportion angular waste	7.8	15.2	18.7
	Proportion chips	18.2	6.1	13.7
	Proportion retouched tools	0.2	16.6	1.5
	Proportion backed tools	0.0	10.2	0.0
	Proportion utilized tools	0.9	14.6	2.3
Artifact conditions	Proportion non-obsidian raw material	39.5	3.5	2.7
	Proportion broken blanks	44.6	10.2	44.3
	Proportion burnt artifacts	0.0	0.3	0.2
	Proportion cortical artifacts	15.2	28.9	18.7
Artifact ratios	Blank/core ratio	31.5	5.7	75.8
	Tool/core ratio	0.8	4.8	1.8
	Tool/blank ratio	0.0	0.8	0.0
	Flake/bladelet ratio	77.8	1.1	1.0
	Retouched/utilized ratio	0.3	1.1	0.6
Core characteristics	Proportion freehand cores	80.0	52.6	100.0
	Proportion bipolar cores	20.0	47.4	0.0
	Proportion cortical cores	70.0	0.1	75.0
Blank attributes	Proportion convergent edge contour	42.1	5.7	4.3
	Proportion oval edge contour	31.6	12.8	19.7
	Proportion straight & parallel edge contour	16.3	74.8	62.5
	Proportion rejuvenation/debordant flakes	10.3	16.1	18.7
	Proportion unidirectional dorsal negatives	82.4	51.9	95.9

#### Core reduction and management

At all three sites, cores are exclusively made from obsidian. The Dimtu cores exhibit a size variability that encompasses the full range seen across the assemblages (21.4 ± 6.9 mm length, 17.9 ± 4.3 mm width, 14.7 ± 5.0 mm thickness; Fig. 9A–C). High standard deviations, particularly in length, reflect notable variation in core dimensions, although the small sample size (N = 10) might influence this. A key feature at Dimtu is the absence of bladelet cores; no bladelet reduction negatives were identified on any core, correlating with the absence of bladelets. The cores display high levels of exploitation, using either opportunistic reduction methods with multidirectional removals, e.g., on a core-on-flake (Fig. 10: ID 1540), or unidirectional reduction using a cortical platform (Fig. 10: ID 1555). Platform preparation is absent, and the number of visible detachments ranges from four to eight flake removals per core. Two smaller cores show clear signs of bipolar flake reduction. Simbero’s core assemblage (5.5% of total) is the largest proportion found, with cores being smaller than those at the other sites (18.0 ± 3.1 mm length, 13.6 ± 2.8 mm width, 10.6 ± 2.1 mm thickness; Fig. 9A–C). Prismatic and pyramidal cores are mainly used for a unidirectional reduction of flakes and bladelets, with both core types featuring prepared platform surfaces, often from prior flake removals (Fig. 10: ID 658, ID 768, ID 782). These cores show up to 14 negative scars and are regularly maintained with rejuvenation and debordant flakes (Table 5: 16.1%; N = 9). The small dimensions of the cores indicate intensive exploitation and high standardization. The sizes of bipolar cores at Simbero suggest that the use of the anvil technique occurred as a terminal stage of core reduction. The Webi Gestro core sample is small (N = 4), making dimensional comparisons of these largest cores in all three assemblages difficult (26.9 ± 2.2 mm length, 15.2 ± 2.6 mm width, and 15.8 ± 4.6 mm thickness). However, the limited number of cores, combined with the presence of high quantities of cortical remains, corresponds to typical nodules found near the local Wasama outcrop (Table 5; Fig. 10: ID 60), suggesting that many cores were likely exported after brief use at the site. Two pyramidal bladelet cores show unidirectional reduction from a single, prepared platform (Fig. 10: ID 75, ID 163). In contrast, a third core exhibits a mixed reduction strategy including rotation with both flake and bladelet negatives from multiple encircling platforms (Fig. 10: ID 60). There are no bipolar cores in the Webi Gestro assemblage.

#### Blank production

The Dimtu lithic assemblage clearly focuses on flake production, with flakes making up 69.7% of the total assemblage (Fig. 8). Consequently, its flake-to-bladelet ratio is more than 70 times higher than in the other two assemblages (Table 5). Metric analysis of fully preserved blanks shows average dimensions of 28.6 ± 5.7 mm in length, 18.0 ± 3.3 mm in width, and 5.3 ± 3.4 mm in thickness (Fig. 9A-C). Except for a few outliers, no clear transitional range toward bladelets is visible (Fig. 9G). Although most flakes are relatively large, the elongation index is the lowest among all sites studied (Fig. 9J), indicating the production of comparatively wide (Fig. 9E), thick (Fig. 9F), and consistently pointed flakes – a specific trait of this assemblage, despite their rarity (n = 19; 6.1% of all flakes). Blanks thus exhibit mainly convergent (42.1%) or oval (31.6%) edge contours. Flakes longer than 30 mm are regularly present (Fig. 8). Flake widths always match the widths of the negatives on the assemblage’s cores, but their lengths do not correspond to core lengths. This hints to the intensive exploitation of cores at the site, also supported by the high blank to core ratio (Table 5). The already mentioned presence of bipolar cores is reflected in identifiable bipolar flakes and the highest proportion of chips in the dataset (18.2%; Fig. 8). The large share of broken flakes (Table 5: 44.6%) likely results from the intensive use of bipolar technology, compounded by the predominance of lower-quality raw material (Table 5). Flake platforms mainly show natural fissures or cortical surfaces (Fig. 11: ID 1580), with only a single finely facetted platform recorded (Fig. 11: ID 1600). The dorsal scar patterns indicate exclusively unidirectional reduction along distal and lateral convexities, further supported by 32 rejuvenation flakes (Table 5: 10.3%). Blank production at Simbero is characterized by a balanced ratio of flakes to bladelets (Table 5), with both blank types produced in nearly equal proportions (Fig. 8). The metric dimensions of complete blanks average 22.8 ± 5.6 mm in length, 11.1 ± 4.2 mm in width, and 4.6 ± 4.0 mm in thickness (Fig. 9A–C). In terms of the general shape, straight and parallel edges (74.8%) clearly predominate at this site, regardless of whether flakes or bladelets are considered. The length/width biplot (Fig. 9H) reveals no clear separation between flake and bladelet values, suggesting a technological continuum between both categories. This somewhat contradicts the earlier observation of strict blank production from either flake or bladelet cores, pointing instead to a more flexible reduction strategy. Interestingly, although Simbero features the smallest average core dimensions (Fig. 9A–C), its blank dimensions are not the smallest (Fig. 9D–F). This suggests that cores were exploited to a high degree, while blanks from earlier reduction stages were regularly discarded. Supporting this, the assemblage contains the highest proportion of cortical blanks (Table 5), indicating repeated decortication during reduction. Another distinctive feature is the comparatively low percentage of fragmented blanks (Table 5), which may reflect both a more careful reduction approach and the extensive use of high-quality obsidian (Table 5). Products of bipolar reduction (flakes and bladelets) occur regularly in the assemblage. Notably, Simbero exhibits a low blank-to-core ratio (Table 5) despite the presence of highly reduced cores. This may imply that blanks produced here were exported from the site and/or that cores arrived already partially reduced – possibilities that cannot be definitively confirmed or ruled out. A key feature of the Webi Gestro assemblage is the high percentage of blanks. While the share of flakes (31.2%) and bladelets (31.8%) are almost identical and exhibit a balanced flake/bladelet ratio (Table 5), the share of bladelets is notably the highest of all investigated sites (Fig. 8). The blank-to-core ratio (Table 5: 75.8) is more than 13 times higher than at Simbero but rather indicates the low number of cores. Indicated by the negatives on the dorsal faces, unidirectional reduction is clearly dominating (95.9%), in tandem with mostly parallel edge contours (62.5%) of the produced blanks (Table 5). The average length is 20.3 ± 5.4 mm, width 7.6 ± 3.7 mm, and thickness 2.4 ± 1.7 mm. Compared to the other sites; metric data confirm considerable differences in the flakes/bladelets produced here in terms of their size dimensions and their even smaller standard deviations. Some bladelets (N = 20) even have a needle-like shape with exceptionally small width and thickness (Fig. 11: ID 867). This is also corroborated by the highest elongation index of blanks at Webi Gestro (Fig. 9J). The high proportion of more than 40% broken blanks (Table 5) might be related to the production of such delicate bladelets. The highest share of angular waste (Fig. 8), on the contrary, is, however, related to non-obsidian materials. Analogous to the lack of bipolar cores, bipolar flakes/bladelets are entirely lacking; this might indicate that cores were not reduced to a maximum but likely exported. Fittingly, a comparatively large number of flakes (Table 5: 18.7%; N = 28) can be assigned to core preparation and maintenance function (debordant and rejuvenation flakes).

#### Tool manufacture

While the pointed flakes of the Dimtu assemblage might be interpreted as intended end products, there is no clear standardization in size or shape (Fig. 11). Moreover, evidence of retouch is absent in the Dimtu assemblage. Merely, occasional signs of use are visible to the naked eye, such as edge damage on individual points (Fig. 11: ID 1598, ID 1626) or wear on both ventral edges caused by use (Fig. 11: ID 1580), alongside other less distinct indications of flake utilization. Correspondingly, the assemblage exhibits the lowest tool-to-core and tool-to-blank ratios (Table 5), the latter being especially notable given the exceptionally high number of blanks. Only Simbero has yielded a significant number of retouched, backed, and utilized tools, making the presence of these components a characteristic feature of its assemblage composition (Fig. 8). The tool-to-blank ratio is at least 36 times higher than at the other sites (Table 5), pointing to the importance of tool manufacture at Simbero. Considering the backed tools, points of impact are observed to be located on the proximal end of the tools (n = 4), while a transversal striking direction also occurs (n = 2). Analysis of the backed tools indicates that impact points are regularly identifiable on the proximal ends (n = 4), with evidence of transversal striking in a few cases (n = 2). Although the sample size is too small for definitive conclusions, the data nonetheless support the hypothesis that straight-backed tools (Fig. 11: ID 755) were produced on *bladelets*, whereas transversal flaking was applied to *flakes* to create curved-backed implements (Fig. 11: ID 520): In the depicted orientation, the original flakes were struck transversely to the tool axis. The former striking platforms were then removed during backing, while the bulbs of percussion were preserved in two cases on the ventral face of the former flake, indicating the transversal striking direction. Mean values of backed tools (N = 35) are 20.8 ± 5.3 mm (length), 7.2 ± 1.5 mm (width), and 4.2 ± 0.9 mm (thickness). Low standard deviations, as well as the total absence of outliers (Fig. 9K), hint at a high degree of standardization in terms of the size of these tools. Some standard mean ratios have additionally been calculated for the backed tools (elongation: 3.0 ± 1.0 mm, robustness: 1.8 ± 0.5 mm, relative thickness of the back: 1.0 ± 0.2 mm). While we refrained from subcategorizing backed tools here, it is clear that despite the dimensional uniformity, a range of diversity is captured in the current sample in terms of morphology, as well as location and method of backing (Fig. 11). Apart from backed tools, other retouched pieces are restricted to various scraper forms. Many signs of utilization occur on unretouched artifacts, mostly on bladelets (82.3%), and hint to the fact that use of these products played an important role at this site. At Webi Gestro, most blanks remained unretouched (Fig. 11: ID 59, ID 867). Exceptions occur only on bladelets: a single specimen shows fine retouch – not to be confused with backing – of its entire right lateral edge (Fig. 11: ID 8). Single notches, mostly restricted to one edge of bladelets, form the only and unique characteristic tool at Webi Gestro, noted at least on six artifacts. Notching is both observed on the ventral (Fig. 11: ID 880) and the dorsal face (Fig. 11: 850), in one instance restricted to the edge (Fig. 11: ID 58). The only connecting factor is the position in the medial part of the bladelets.

### Use and function of lithic tools

The preliminary functional analysis of the Simbero (n = 7) and Webi Gestro (n = 5) samples revealed an overall good preservation of microwear. Possible functional residues were detected on three tools from Simbero and two tools from Webi Gestro. These initial results demonstrate the high potential of the assemblages for further functional studies. The seven pieces in the Simbero sample showed good (n = 3), moderate (n = 2) and moderate to poor (n = 2) states of surface preservation when examined under low and high magnification. The sample dominated by backed pieces (n = 6), revealed the use of these artifacts as projectiles (one certain identification and three tentative). The backed artifact providing the most explicit evidence presented a scar pattern suggestive of oblique hafting (Fig. 12; cf. Goldstein and Shaffer 2017: fig. 10), but this interpretation requires experimental testing with close morphological replicas hafted in different configurations, as previous experiments with obsidian crescents have reported overlapping damage patterns from different hafting orientations and concluded that the existing reference sets are too small for reliable distinctions (Goldstein and Shaffer 2017: table 2). One of the possible projectiles (ID 740) had black residue on its ventral proximal surface that was interpreted as charred organic material on visual inspection and might relate to hafting (**Fig. S2**), although possible incidental nature needs to be carefully ruled out in future analyses (cf. Schmidt et al. 2015). The distribution of the residue is inconclusive with respect to the hafting orientation of the armature. Another tentative projectile (ID 755) showed a black residual deposit on its ventral distal tip for which a natural origin could not be ruled out at this stage. In addition to projectile armatures, the sample contained knives (one certain identification and one tentative). The last artifact (ID 561) in the sample displayed limited wear that could match with either knife or projectile use. The edge scarring was associated with parallel linear features, but remaining surface deposits prevented a full evaluation of the extent of the microwear, which would be necessary to make the distinction. The knife with the most abundant microscopic use-wear (ID 761) additionally showed linear features along the backed edge that could relate to hafting, but experiments are needed to rule out other explanations, including taphonomy (Fig. 13). A tentative knife presented a linear deposit of similarly charred material on both aspects of the working edge that by its distribution and appearance could represent use residue transformed by heat (Fig. 14). It is worth emphasizing, however, that visual criteria have limited reliability in determining the composition and origin of archaeological stone tool residues (Croft et al. 2018; Douze et al. 2020; Kozowyk et al. 2020), and all these remains therefore require future chemical analyses for full characterization. The Webi Gestro sample (n = 5) showed good (n = 1), moderate (n = 3) and poor (n = 1) states of surface preservation. The sample comprised two fragmentary bladelets with evidence for transverse use of lateral edges. ID 534 is a chert blade fragment that showed unilateral wear with hide-like characteristics (Fig. 15). Red iron oxide residue was recorded dispersed over the surfaces of the tool without clear patterning and provisionally interpreted as deriving from the burial environment, given the consistent presence of iron oxide in the stratigraphy, which accounts for 9–10% of the layers in question (B. Glaser/T. Bromm, pers. comm.). Well-developed use-wear is restricted to the right lateral edge of the tool, and the left edge only shows limited scarring that may derive from hafting or prehension. Determining the contact material for the second artifact, a minimally retouched obsidian bladelet fragment (ID 535, Fig. 16), awaits experimental replication. The use-wear is present on both lateral edges, with the left edge showing signs of resharpening. A narrow, proximally curved bladelet in obsidian from Webi Gestro (ID 528) showed scarring and linear microwear features on both lateral edges. The left edge has long linear features probably related to knife use. The opposite edge has linear wear in the proximal extremity that is strongly developed but different from the striations on the left edge, and experiments would be helpful in investigating whether these features (Fig. S3d) could be linked to hafting. Haft wear has been previously recorded on experimental obsidian tools used in chopping wood (Kononenko et al. 2015) and adapting experimental protocols to the Bale Mountains lithic samples can be recommended for further interpretation of the trace patterns. If the wear on both lateral edges of ID 528 instead derives from use, the observed differences would indicate two or more worked materials.The remaining two artifacts in the Webi Gestro sample bore less informative wear traces. ID 525 had limited bending-initiated scarring that could derive from projectile or (brief) knife use, but poor surface preservation prevented further high magnification analysis. ID 527 displayed one location on each lateral edge with limited, transversely oriented traces for which a taphonomic origin could not be ruled out in preliminary analysis. Notably, both artifacts showed residual deposits on their ventral distal tips. On ID 525 the residue was white in color and high in carbon (Fig. S4). ID 527 presented a transparent deposit equally rich in carbon that could represent lipid residue (Fig. S5). At this stage, neither of the deposits can be linked to tool use or hafting due to the limited development and/or preservation of use-wear. Preliminary functional data for the Simbero and Webi Gestro samples is summarized in Table 6.

**Table 6:** Summary of use-wear and residue observations for the Simbero (n = 7) and Webi Gestro (n = 5) lithic samples

Artifact ID	Site	Tool class	Use-wear interpretation	Surface preser-vation	Residue description	Inferred residue cause (confidence level 0–4)
525	Webi Gestro	Utilized bladelet	Projectile or knife	Poor	White residue on ventral distal surface near tip, high in C, possibly ash or bone	Use (0)
527	Webi Gestro	Utilized bladelet	Use not confirmed	Moderate	Transparent residue on ventral distal surface, high in C, possibly lipid	Use (0)
528	Webi Gestro	Utilized bladelet	Knife	Good	-	-
534	Webi Gestro	Utilized bladelet	Transverse motion	Moderate	Red residue dispersed over tool	Environmental (3)
535	Webi Gestro	Utilized bladelet	Transverse motion	Moderate	-	-
537	Simbero	Backed tool	Projectile	Good	-	-
538	Simbero	Backed tool	Possible knife	Moderate	Black residue on active edge, charred organic matter?	Use (1)
561	Simbero	Utilized bladelet	Projectile or knife	Moderate	-	-
740	Simbero	Backed tool	Possible projectile	Good	Black residue in ventral proximal part, charred organic matter?	Hafting (2)
754	Simbero	Backed tool	Possible projectile	Moderate	-	-
755	Simbero	Backed tool	Possible projectile	Moderate	Black residue on ventral distal tip (charcoal)	Environmental or hafting (0)
761	Simbero	Backed piece	Knife	Good	-	-

## Discussion

Numerous precise radiometric dates allow for a detailed reconstruction of landscape changes in the high-altitude Bale Mountains. Deglaciation began around 18–17 ka, and initial sedimentation occurred in smaller depressions on the Sanetti Plateau, although the central peak was still ice-covered. By approximately 16 ka, ice started to recede from the western, northern, and eastern valleys and the central Sanetti Plateau, including the Big Boulder moraines near the highest peak, Tullu Dimtu. At the same time, sedimentation commenced at Lake Garba Guracha, the region’s largest basin. Around 15 ka, this drastic transformation of the landscape was accompanied by a rapid shift to warmer and wetter conditions, coinciding with the reoccupation of the Bale Mountains by hunter-gatherer groups. Although likely brief, this occupation took place within a significantly expanded Afroalpine environment and included the use of sites both along the northern and western escarpment and on the recently deglaciated Sanetti Plateau.

All stages in the lithic reduction processes in the three sites’ assemblages analyzed here show significant differences, however, partly resting on small sample sizes. These differences are most evident in the distinct provisioning strategies for local raw materials (obsidian vs. chert, secondary [rounded nodules] vs. primary sources). At Simbero, the standardized backed tools were predominantly made from locally available chert, whereas imported obsidian from unknown sources at Webi Gestro and Dimtu indicates that lithic production strategies were flexibly adapted to both local and regional raw material availability. In terms of core reduction, differences become clear in terms of the preparation and maintenance strategies, the metric dimensions, the degree of exploitation, striking direction, preferred blanks, and the presence/absence of the bipolar technique. In terms of blank production, the flake/bladelet ratio observed at Simbero and Webi Gestro might be regarded as the only similarity. However, apparent differences exist in the respective metric values and their general shape (e.g., edge contours). In fact, at each site, one specific blank can be isolated: relatively thick pointed flakes at Dimtu (albeit rare), short blanks in the flake-bladelet transition zone at Simbero, and comparably elongated bladelets at Webi Gestro. Likewise, tool manufacture also witnesses significant differences, with Simbero blanks being transformed into various backed tools and scrapers. In contrast, at Dimtu, retouch is entirely missing, while the assemblage from Webi Gestro features medially notched bladelets. One unretouched bladelet from the latter site exhibited ash or bone residue, with limited microwear pointing to its use as a projectile or knife. Another specimen showed potential lipid residue confined to a single edge, though it lacked well-preserved use-wear traces. Several other (fragmented) bladelets demonstrated use as knives, showing wear patterns consistent with transverse, oblique, and longitudinal motions. In contrast, at Simbero, organic residues were more widespread on backed tools. These included possible adhesive remains and charred material, likely related to hafting and use. Although the sample size is limited, the backed tools from Simbero indicate functions as both projectile elements and knives. Future experimental studies adapted to tool morphologies and raw materials documented at the Bale Mountains sites, alongside with in-depth chemical analyses of the stone tool residues, would enable further insights into tool use activities, site function, and hafting arrangements. In the Ethiopian context, functional studies are still infrequently integrated into lithic analyses, with some results, such as those from Mochena Borago, remaining unpublished to date.

The technological flexibility observed in the aforementioned production stages and the resulting assemblage compositions is noteworthy. Considering the varying site locations and the limited temporal scope addressed in this study, these differences likely reflect variability in site function and associated mobility strategies. However, due to the small sample sizes – particularly regarding the functional analysis – it is not possible to reliably reconstruct site-specific activity patterns. The following observations should therefore be regarded as provisional and may primarily serve to inform future research: At Dimtu, the rare occurrence of unretouched pointed flakes, hardly ever bearing macroscopically visible signs of use, may indicate that these were mostly manufactured without the intention of immediate use at the site, potentially to be carried along in order to finish them at different locations. This inferred pattern of behavior, coupled with the absence of cores and the predominant use of Kore chert from the Genale Plateau (Fig. 1C), may indicate an efficient use of raw materials within a highly mobile, Bale-wide land-use system. It may also reflect planned hunting activities on the Sanetti Plateau, which – based on the evidence presented here – had become ice-free during this period and was dominated by expansive open Afroalpine vegetation. These environmental conditions likely attracted larger mammal species, thereby offering favorable circumstances for sustained or strategically organized hunting. At Simbero, the backed tools exhibit similar elongation values but differ notably in having lower overall robustness and a higher relative thickness of the back, compared to broadly contemporaneous backed tools from the lowland MER. While this pattern is consistent with the considerable variability typically observed among Late Glacial backed tools (Leplongeon et al. 2020b), the low standard deviations across all dimensions of Simbero’s backed tools may simply reflect small group sizes (e.g., Liu et al. 2020). At Webi Gestro, the export of cores and occasional manufacture of uniquely notched bladelets hint at a transient site function. Fishing activities should be explored given the location of the site, although currently direct evidence of fishing in the form of fish remains is missing. This leads to other – albeit unifying aspects observed at all sites: the general lack of faunal remains despite good organic preservation, the weak signs of burning activities that might indicate food preparation and consumption, the overall relatively low density of finds, and the concentration on the production of few characteristic tools or even blanks – often highly standardized – point to small groups and short visits at the sites. Marked differences in burning-related features and faunal quantities indicate that site use during the African Humid Period was less intensive than in later Holocene occupations. Artifact densities are low (12–18 tools per m^2^ at Dimtu and Webi Gestro), corroborating the limited faunal remains and sparse evidence of burning. In contrast, Holocene layers show higher faunal abundances and more frequent burning features, consistent with more sustained occupation. Together, these patterns reflect temporal shifts in settlement intensity and activity organization in response to changing local environmental conditions. These relationships will be examined in greater detail in a forthcoming study integrating archaeological datasets with soil biogeochemical proxies. 

Likewise, distinct differences emerge that set the archaeological record of the early AHP in the Bale Mountains apart from that of earlier Late Pleistocene MSA hunter-gatherers during the *local* Last Glacial Maximum (LGM). The latter is characterized by the presence of a residential site and indications of extensive logistical mobility, the firm reliance on local obsidians, the massive accumulation of cultural material, and a relatively enduring integration of high-altitude landscapes into the land use system of mobile foragers (Ossendorf et al. 2023). Finally, the preparation and consumption of staple food during the MSA occupation (Ossendorf et al. 2019) forms another relevant distinguishing factor. The hypotheses that the Bale Mountains’ early AHP occupation was of explorative character (maybe not even sufficient to meet the high-altitude challenges in the long term) might be allowed for future investigations to be verified. This interpretation is supported by the observation that contemporaneous early AHP occupations in the Horn of Africa’s lowlands persisted somewhat longer, extending up to the onset of the Younger Dryas (Leplongeon et al. 2020a). In contrast, the stratigraphic sequences examined in the Bale Mountains reveal no clearly distinguishable cultural deposits that could be attributed to the subsequent phases of the early AHP, the Younger Dryas, or the Early Holocene. The limited Early Holocene dating results are not associated with archaeological assemblages but only with a few non-diagnostic lithic artifacts, suggesting only sporadic human use of the Bale Mountains.

Turning to the regional scale, it is essential to note that during the early AHP, human networks extended to high altitudes. Electron microprobe results presented here show that only one single specimen from Webi Gestro (Bale 043) is likely to reflect local procurement at the Wasama outcrop. In contrast, some samples (Bale 066, 068, 086) were imported to the Bale Mountains from unknown sources, even out of the range of currently known geochemical compositions. The majority of the remaining samples cluster in terms of their values between those of several MER volcanoes and the Wasama outcrop. Direct distances from the Bale Mountains to the statistically closest candidates amount to at least 170 km (Worja), 190 km (Artu 3/4), 200 km (Abadir 2/3, Guddo), and 270 km (Assebot). Interestingly, no matches could be observed with values of obsidian sources in the central MER, which are closer to the Bale Mountains, such as the Chebe or Korbeti volcanoes (140 km). In total, this instead suggests that the majority of samples originate from currently unknown obsidian sources. There is no means to estimate whether these were closer to the Bale Mountains or to the sources mentioned above. Notably, the early AHP samples display less pronounced statistical similarities with the local Wasama outcrop when compared to the MSA samples, which exhibit stronger and more consistent geochemical matches with the local source. However, the fact that most of the above samples’ values scatter between the mentioned MER volcanoes is rather a hint to a broader region of origin for these obsidian artifacts, instead of a restricted single source. Importantly, early AHP hunter-gatherers shared this larger region, as obsidians from all three Bale sites analyzed here belong to this scatter. This, in turn, has two implications: firstly, it suggests that the early AHP occupations at the three investigated sites formed a coherent settlement episode. Secondly, it indicates extensive exchange and cultural transmission within the larger region. However, it is important to emphasize that these geochemical patterns do not permit definitive source assignments or detailed mapping of exchange networks. The existence of large and widely spanning networks formed a prerequisite for the occupation of the Bale Mountains. Recently published dating results point to early AHP human presence in the southwestern Ethiopian highlands at Sodicho (Hensel et al. 2021). According to a review by Leplongeon (2020a), several early AHP occurrences in the central MER region and beyond are characterized by a diversity of production concepts. While primarily based on bladelet production, a striking diversity in the number of retouched tools is evident, with backed tools not necessarily present in the respective assemblages. The existence of more versatile subsistence strategies is based on the larger functional variability observed and includes fishing. Ménard et al. (2014) report a”one tool per site” phenomenon at slightly younger sites in the Ziway-Shala basin of the central MER. Given this, the authors correctly predicted that only part of the coeval technological variability is currently known. To explain the above phenomenon, the authors proposed either subsistence specialization or rapid demographic transformations. With the evidence of the Bale Mountains adding to the variability of early AHP occurrences in the Horn of Africa, we argue for the former: the limited time span documented in the Bale Mountains offers a unique snapshot of contemporaneous behavior, showing simultaneous, site-specific use of raw material sources and associated technological strategies. Despite the clear links between both regions, it has to be emphasized that high-altitude dwellers did not simply import “lowland strategies”, but also developed their own independent local, on-the-ground solutions, visible, e.g., in the specific assemblage compositions and in the pointed flakes from Dimtu, for which there are no contemporaneous equivalents.

Finally, this early AHP occupation represents a second Late Pleistocene settlement phase, characterized by simultaneous high-altitude and lowland occupation, at least in the regions considered here (Ossendorf et al. 2023). These synchronous occupations occurred irrespective of environmental conditions, as no indication of unfavorable, stressful lowland conditions can be observed during either of the phases. Nor did strict ecological "envelopes" exist at high altitudes, as occupations in the Bale Mountains occurred during maximum glaciation, as well as after deglaciation during warmer and wetter conditions of the early AHP (the same pattern can be observed during both wet and dry phases in the Holocene: Tekelemariam 2021). The concept of refugia, apart from additional definitional problems, is not likely to capture these patterns in the archaeological record. Instead, in Ethiopia, we observe phases with high visibility in several ecozones and landscapes probably connected to population expansions and facilitated by the establishment and maintenance of extensive social networks. These networks were multiscale, integrating high-altitude landscapes by allowing for vertical landscape exploitation (Vogelsang and Wendt 2018) without requiring the permanent residence of mobile groups in the challenging highest altitudes. Diachronically, these phases are replaced by periods of low visibility, with little to no archaeological evidence in both high- and lowlands. The latter may be due to people retreating into currently unknown regions and a reduction in the extent and density of networks. However, the increased research efforts of recent years also point to research bias as a simple cause (see also Leplongeon et al. 2023, 2025). Knowledge about human presence in various ecozones of the Horn of Africa has been considerably expanded by newly discovered and investigated sites (e.g., Schepers et al. 2020; Hensel et al. 2021; Niespolo et al. 2021; Ashkenazy and Sahle 2021; Sahle et al. 2025), accompanied by the reanalysis of the chronology and material from several sites (e.g., Gossa et al. 2012; Jones et al. 2018, 2021; Reid et al. 2019; Sahle et al. 2024). To move forward, we propose placing greater research focus on these ecozone-transcending networks. Similarities in material culture and behavioral implications demonstrate a significant overlap in expansion phases, despite differences in ecology, and indicate greater interconnectedness among populations. Provided that standardization of archaeological data progresses (postulated e.g. by Fusco et al. 2025), it will be crucial to develop means to measure the interconnectivity between forager groups as expressed through their material record at various scales. Identifying diachronically fluctuating networks would also reveal the deliberate, collective choices of prehistoric hunter-gatherers.

## Conclusions

This study demonstrates that, during the early African Humid Period (AHP), hunter-gatherer groups deliberately incorporated the deglaciated high-altitude Bale Mountains into their broader subsistence and settlement systems, despite the ecological constraints inherent to such challenging landscapes. The concurrent use of three distinct sites was accompanied by diverse raw material provisioning strategies and notable technological flexibility – likely reflecting task-specific organization and adaptive responses tailored to short-term occupations by small, mobile groups across varying Afroalpine landscape units. The initial functional insights presented here highlight these dynamics, and future analyses based on larger samples hold considerable potential to further refine and expand our understanding. Notably, these high-altitude communities were part of extensive networks, which enabled strong interregional connectivity and cultural transmission, though precise exchange pathways remain unresolved. These findings challenge previous assumptions that high-altitude regions either represented human environmental refugia or were marginal, with only sporadic use during the Late Pleistocene. Moreover, even when considering the broader Ethiopian Highlands as a potential refugial zone, our data suggest that early AHP human groups actively exploited these landscapes as part of wider settlement and subsistence strategies rather than retreating solely to refugia. For future research, the development of comparative frameworks and standardized approaches to data collection and inter-site comparison will be essential to further explore past collective decision-making processes, as expressed through the shifting interconnectivity of prehistoric networks.

## Supplementary Information

Below is the link to the electronic supplementary material.

Supplementary file1 (DOCX 2369 KB)

## Data Availability

The authors confirm that all data generated or analysed during this study are included in this published article and the supplementary materials. Furthermore, primary and secondary sources and data supporting the findings of this study were all publicly available at the time of submission. Artifact and faunal collections are curated at the National Museum of Ethiopia, Addis Ababa.
