# Supplementary material for: Human occupation of the Afroalpine Bale Mountains at the onset of the African Humid Period
Source: Landsc Ecol. 2026 Apr 9;41(4):75. doi: 10.1007/s10980-026-02337-8 (PMC13083501; doi:10.1007/s10980-026-02337-8)
Supplement: Supplementary file 1 — Supplementary file1 (DOCX 2369 KB) [file 10980_2026_2337_MOESM1_ESM.docx]

**Supplementary Material for:**

**Human Occupation of the Afroalpine Bale Mountains at the Onset of the African Humid Period**

Götz Ossendorf*, Minassie Girma Tekelemariam, Noora Taipale, Alexander R. Groos, Agazi Negash, Dries Cnuts, Naki Akçar, Christof Vockenhuber, Zinash Kefyalew Tariku, Trhas Hadush Kahsay, Veerle Rots, Ralf Vogelsang

*Corresponding author. Email: gossend1@uni-koeln.de

**This file includes:**

Supplementary Stratigraphic Descriptions

Tables S1 to S5

Figs. S1 to S5

**Supplementary Stratigraphic Descriptions**

This section provides detailed information for each Bale Mountains site, including location, environmental context, stratigraphy, and preliminary archaeological interpretations. **Figs. 1–3** provide additional context, illustrating site locations, topography and stratigraphic sequences, while Supplementary **Tables S1–S2** summarize stratigraphic units, sediment characteristics, radiocarbon dates, and associated archaeological materials for Dimtu, Simbero, and Webi Gestro.

**Dimtu**

**Location and context:** The Dimtu rock shelter (6.80°N, 39.80°E; 4,023 m asl) is a previously undocumented archaeological site on the central Sanetti Plateau of the Bale Mountains, approximately 2 km from Tullu Dimtu. The site comprises several small rock shelters embedded in a weathered basalt ridge, occupying an exposed high-altitude plateau setting characterized by cold, dry conditions, strong winds, and pronounced temperature fluctuations. The shelters’ orientation provides partial protection from prevailing trade winds, contributing to locally stable depositional environments. A single test excavation of one square meter in the uppermost shelter revealed a well-stratified sedimentary sequence preserving two archaeologically distinct occupation phases.

**Stratigraphy:** The Dimtu rock shelter preserves a stratified sedimentary sequence resting directly on bedrock and comprising three stratigraphic units (SU I–III).

SU I (GTS) forms the uppermost deposit and is a thin (0–2 cm), laterally continuous layer with a sharp basal contact. The sediment consists of dry, loosely packed, poorly sorted silty sand with angular clasts and a yellowish-brown color (5Y 6/4). Sedimentary structures are absent. Charcoal occurs only in trace amounts, while ash, bones, artifacts, and other organic remains are lacking. Minor roof fall and sparse root intrusions are present. The unit represents a largely sterile surface deposit, likely formed through aeolian input and minor frost-related reworking.

SU II (BAC) is 8–21 cm thick and displays a diffuse lower boundary and slightly inclined bedding. It is composed of dry, loose, well-sorted silt with angular clasts, showing a color range from very pale gray/white (N 9.5/1) to very dusky red (2.5R 1/2). Laminated ash and charcoal horizons are present. Charcoal, ash, bones, and lithic artifacts occur throughout the unit. Hearths, burnt sediments, trampling, and repeated occupation surfaces indicate intensive anthropogenic activity. Two radiocarbon determinations place this unit in the Late Holocene (1.7–1.5 ka cal. BP).

SU III (DBR) directly overlies bedrock and forms the basal unit of the sequence (12–25 cm thick). The sediment consists of dry, moderately compact, poorly sorted, slightly gravelly sand with angular clasts and a light yellowish-brown color (10YR 6/3). Charcoal, ash, bones, lithic artifacts, and organic material are evenly distributed. Clear traces of fire use and trampling are present, while thin carbonate crusts indicate minor post-depositional chemical alteration. Radiocarbon ages between 14.9 and 13.8 ka cal. BP place this occupation in the Late Glacial.

**Preliminary interpretation:** At Dimtu, the stratigraphic sequence reflects predominantly *in situ* sediment accumulation under cold and dry high-altitude plateau conditions, punctuated by episodic anthropogenic activity. Only limited sediment transport could be observed and mainly include aeolian input, minor frost-related reworking, roof fall, and localized carbonate crust formation. There is no evidence for water reworking or significant downslope displacement, consistent with the site’s exposed plateau setting distant from active drainage systems.

Archaeological signals are confined to SU II–BAC and SU III–DBR, where hearths, charcoal, ash, trampling, and lithic artifacts document repeated occupation events. The basal SU III records Late Glacial activity (14.9–13.8 ka cal. BP), followed by a substantial temporal gap and renewed, short-term occupation during the Late Holocene in SU II (1.7–1.5 ka cal. BP). The largely sterile SU I represents a thin surface deposit formed under recent high-altitude environmental conditions. Overall, Dimtu preserves a high-integrity, stratified record of intermittent human presence within a stable depositional environment.

**Simbero**

**Location and context:** The Simbero rock shelter (7.01°N, 39.74°E; 3,520 m asl), originally reported by Reber et al. (2018) as “Rock Shelter 1”, is located on the northwestern escarpment of the Bale Mountains, approximately 50 m above the Simbero Valley and overlooking the upper Web Valley. The escarpment setting is characterized by comparatively humid conditions, enhanced sediment moisture, and increased biological activity. Test excavations covering two square meters exposed a dense, well-stratified archaeological sequence with abundant lithic and faunal remains, documenting repeated, though discontinuous, occupations from ca. 15 ka cal. BP into the recent past.

**Stratigraphy:** The Simbero rock shelter preserves a dense, well-stratified archaeological sequence resting directly on bedrock and comprising five stratigraphic units (SU I–V), reflecting repeated occupation under persistently moist escarpment conditions.

SU I (LSG) forms a thin (2–6 cm) upper deposit with diffuse boundaries and inclined bedding. The dry, loosely packed, well-sorted silty–sandy sediment is pale yellowish brown (10YR 6/6) and contains angular clasts. Charcoal occurs only as isolated fragments and ash is absent, while organic material (roots, wood fragments, animal excrement) is common. Artifact densities are low, with few lithic and ceramic finds. Evidence for hearths, burnt sediments, and trampling indicates light but recurrent recent activity, strongly overprinted by biological inputs.

SU II (SCG) is highly variable in thickness (2–35 cm) and sharply bounded at the base. The slightly moist, very strongly compacted, poorly sorted sandy sediment is very dark grayish brown (10YR 3/2) and heavily influenced by decomposed dung. Abundant dung accumulations, mixed fabrics, and phosphate crust formation indicate intensive anthropogenic modification. Charcoal is absent, while bones and few artifacts, including glass beads and lithics, are present. Radiocarbon dating indicates a modern age.

SU III (GCB, LDO, LCB) comprises three stratigraphically related layers reflecting complex depositional dynamics under moist conditions.

- Layer GCB (7–45 cm) consists of wet, slightly compacted, poorly sorted clayey–silty sediment of dark brown color (10YR 3/2). Charcoal, ash, bones, and abundant lithic artifacts are present. Hearths, burnt sediments, and trampling indicate intensive Late Holocene occupation dated to 2.3–2.2 ka cal. BP.
- Layer LDO (0–18 cm) occurs patchily and interdigitates with LCB and GCB. The dry, well-sorted silty–sandy sediment is light olive brown (5Y 6/4). Charcoal is absent, while faunal remains are abundant, and artifacts occur sporadically. Localized carbonate crusts indicate chemical alteration; radiocarbon ages range from 3.2 to 2.5 ka cal. BP.
- Layer LCB (0–15 cm) consists of wet, poorly sorted clayey silt of almost black to very dark brown color (10YR 1/1) and contains abundant charcoal, high lithic artifact density, and bones evenly distributed throughout the unit. Clear evidence for repeated occupation dates this layer to 3.8–3.5 ka cal. BP.

SU IV (LSB) is 7–22 cm thick, sharply bounded, and horizontally bedded. The moderately moist, loosely packed, well-sorted sandy sediment is grayish brown (10YR 5/3). Ash, bones, and isolated charcoal fragments are present, and evidence of fire use and trampling corresponds to Early to Mid-Holocene occupation (8.2–5.1 ka cal. BP).

SU V (CBO) forms the basal unit (0–19 cm thick) resting directly on bedrock. The moderately moist, well-sorted silty–sandy sediment is light yellowish brown (10YR 6/4) and clast-dominated. Charcoal is absent and ash is rare, while bones and lithic artifacts are present. Evidence for repeated fire use and occupation surfaces corresponds to Late Glacial radiocarbon ages (15.0–14.3 ka cal. BP).

**Preliminary interpretation:** The Simbero stratigraphic sequence is strongly shaped by anthropogenic sedimentary and taphonomic processes operating under persistently moist escarpment conditions. Sediment accumulation is largely driven by *in situ* deposition of occupation debris, including charcoal, ash, faunal remains, lithic artifacts, and, in some units, dung accumulations. Natural disturbances are comparatively limited and mainly involve biological activity, localized chemical alteration (including phosphate and carbonate crust formation), and minor reworking of occupation surfaces.

Archaeological signals document repeated and often intensive human occupation from the Late Glacial through the Holocene into the very recent past. The strongest evidence for sustained activity occurs in SU III, where abundant charcoal, lithic artifacts, and faunal remains reflect intensive Late Holocene use. Earlier occupation phases are recorded in SU V (Late Glacial) and SU IV (Mid Holocene), while variability in unit thickness, composition, and artifact density suggests alternating phases of intensified use, reduced occupation, and possible hiatuses. Overall, Simbero preserves a complex, anthropogenically structured sedimentary archive reflecting long-term and recurrent human use of the Bale escarpment zone.

**Webi Gestro**

**Location and context:** Webi Gestro (7.02°N, 39.73°E; 3,423 m asl) is not a rock shelter in a narrow sense, but an overhanging basalt wall running parallel to the Webi Gestro stream on the northwestern escarpment of the Bale Mountains. A narrow terrace at the base of the wall grades downslope into a steep incline approximately 15 m wide, leading directly to the stream channel. This fluvially influenced setting results in persistently moist sediments, enhanced organic preservation, and localized slope-related inputs. Excavations were conducted in two test squares positioned directly adjacent to the basalt wall, spaced 6 m apart, revealing a stratified archaeological sequence previously unreported.

**Stratigraphy:** Webi Gestro preserves a stratified sedimentary sequence developed at the base of an overhanging basalt wall adjacent to the Webi Gestro stream. The sequence comprises five stratigraphic units (SU I–V) reflecting episodic occupation under persistently moist, fluvially influenced escarpment conditions.

SU I (GTS) forms the uppermost deposit and varies between 1 and 7 cm in thickness. It is sharply bounded at the base, inclined, and laterally continuous. The sediment consists of moist, loosely packed, well-sorted silt with a light bluish gray color (5PB 7/1) and a mixed fabric with angular clasts. Sedimentary structures are absent. Roof fall and root intrusions are present, while ash, bones, artifacts, and other organic remains are lacking. Charcoal occurs only as isolated fragments. Minor traces of burnt sediments and trampling without clear hearths suggest short-lived surface activity superimposed on background sedimentation.

SU II (CBG) comprises two stratigraphically coherent subunits reflecting repeated use of a similar depositional substrate.

- The upper CBG subunit (2–20 cm thick) is diffusely bounded and inclined. It consists of moist, compact, poorly sorted sandy sediment of dark reddish gray color (5YR 5/1), clast-dominated, with roof fall present. Large charcoal fragments, minor ash, bones, and lithic artifacts are evenly distributed. Clear evidence of hearths, burnt sediments, trampling, and repeated occupation surfaces indicates intensive anthropogenic activity dated to the very recent past.
- The lower CBG subunit (5–13 cm thick) is sharply bounded and sedimentologically identical. Charcoal is particularly abundant, accompanied by ash, bones, and lithic artifacts. Clear hearths and repeated occupation surfaces are documented. A radiocarbon age of 6.5–6.3 ka cal. BP places this subunit in the Mid-Holocene.

SU III (LRB) is 2–17 cm thick, sharply bounded, inclined, and laterally continuous. The sediment consists of moist, loosely packed, well-sorted clayey silt of red color (2.5R 5/6), matrix-dominated with angular clasts. Roof fall, ash, and charcoal are nearly absent, and only very few bones and artifacts were recovered. The unit reflects a phase of reduced site use, with calibrated age ranges overlapping those of SU II (CBG lower), and is dated to 6.6–6.4 ka cal. BP.

SU IV (SDB, SB) forms a major occupation unit and comprises two stratigraphically related layers.

- Layer SDB (16–24 cm thick) shows a diffuse lower boundary and inclined bedding. The moist, moderately compact, poorly sorted sandy sediment is dark reddish gray (5YR 4/2) and matrix-dominated with angular clasts. Roof fall is common. Large charcoal fragments, ash, abundant bones, and numerous lithic artifacts are evenly distributed. Hearths, burnt sediments, trampling, and repeated occupation surfaces document intensive Late Glacial occupation dated to 14.8–13.9 ka cal. BP.
- Layer SB (2–6 cm thick) is thinner and diffusely bounded, with inclined bedding. The moist, moderately compact, poorly sorted sandy sediment is dark red (2.5R 4/5). Charcoal, ash, bones, and few artifacts are present, but evidence for hearths, trampling, or repeated occupation surfaces is limited, suggesting short-lived activity episodes within the broader SU IV phase.

SU V (LSB) forms the basal unit and reaches up to 29 cm in thickness. It shows diffuse boundaries, inclined bedding, and partial lateral continuity. The moist, moderately compact, poorly sorted sandy sediment is dark reddish brown (5YR 4/3) and matrix-dominated with angular clasts. Roof fall is present. Charcoal occurs only as isolated fragments, while ash, bones, and lithic artifacts are present and evenly distributed. Evidence for hearths and trampling is ambiguous. Radiocarbon ages between 14.8 and 14.0 ka cal. BP indicate Late Glacial occupation broadly contemporary with SU IV.

**Preliminary interpretation:** At Webi Gestro, the stratigraphic record reflects predominantly *in situ* sediment accumulation combined with episodic anthropogenic modification through burning and trampling. The site’s open setting adjacent to the Webi Gestro stream results in consistently moist sediments and enhanced organic preservation, accompanied by localized slope-related inputs. Although inclined bedding and moist conditions are common, there is no clear evidence for extensive water reworking or downslope displacement. Roof fall represents an important natural sedimentary contribution throughout the sequence, while other physical processes, such as minor slope movement and sediment compaction, appear to have had limited impact on overall stratigraphic integrity.

Archaeological signals are strongest in SU II–CBG and SU IV–SDB, where hearths, abundant charcoal and ash, faunal remains, and lithic artifacts document repeated occupation events. Late Glacial activity is recorded in SU IV–SDB and SU V–LSB (14.8–13.9 ka cal. BP), followed by phases of reduced or episodic site use during the Mid-Holocene and renewed very recent activity in the upper CBG subunit. Apparent stratigraphic gaps suggest temporal discontinuities in site use, although their duration and causes cannot be resolved with the current dataset. Overall, Webi Gestro is situated in a hydrologically moist setting, although the sediments show little evidence of fluvial reworking or significant water-driven transport but preserve an internally coherent stratigraphic archive of intermittent human occupation.

**Table S1 Summary of AMS radiocarbon age determinations of charcoal samples from rock shelter deposits in the Bale Mountains discussed in the text.** Calibrated age ranges are reported as 2σ (95.4%) probability intervals in calendar years and were calculated using OxCal v4.4 (Bronk Ramsey 2009) using the IntCal20 calibration curve (Reimer et al. 2020). Sample stratigraphic provenience is documented in **Fig. 3**; site locations are shown in **Fig. 1C**.

| **Site** | **Excavation unit** | **Strati-graphic unit** | **Laboratory Code** | **14C Age (BP)** | **Calibrated Age (max, cal. BP)** | **Calibrated Age (min, cal. BP)** | **δ¹³C (‰)** |
| --- | --- | --- | --- | --- | --- | --- | --- |
| Simbero | O6:NE:L1 | II (CDG) | Beta-552719 | *modern* | - | - | -23.0 |
| Webi Gestro | I4:NW:L2 | II (CBG upper) | COL6832.1.1 | *modern* | - | - | -25.1 |
| Dimtu | C2:NE:L2 | II (BAC) | COL6828.1.1 | 1,715±36 | 1,702 | 1,535 | -22.0 |
| Dimtu | C2:NE:L5 | II (BAC) | COL6829.1.1 | 1,779±36 | 1,742 | 1,569 | -22.9 |
| Simbero | O6:NE:L3 | III (GCB) | Beta-552720 | 2,230±30 | 2,336 | 2,149 | -22.5 |
| Simbero | K4:SE:L9 | IV (LSB) | Beta-610093 | 2,500±30 | 2,727 | 2,469 | -24.4 |
| Simbero | K4:SE:L4 | III (LDO) | Beta-552721 | 2,680±30 | 2,849 | 2,750 | -20.7 |
| Simbero | K4:NW:L4 | III (LDO) | Beta-552722 | 2,900±30 | 3,158 | 2,964 | -23.8 |
| Simbero | O6:NE:L5 | III (LCB) | COL5452.1.1 | 3,337±42 | 3,686 | 3,461 | -25.8 |
| Simbero | O6:NW:L6 | III (LCB) | Beta-610092 | 3,360±30 | 3,689 | 3,488 | -25.5 |
| Simbero | O6:SW:L8 | III (LCB) | Beta-675060 | 3,400±30 | 3,815 | 3,564 | -22.4 |
| Simbero | K4:SW:L8 | IV (LSB) | Beta-552723 | 4,580±30 | 5,445 | 5,055 | -22.8 |
| Simbero | K4:NW:L8 | IV (LSB) | Beta-675061 | 4,660±30 | 5,465 | 5,317 | -22.0 |
| Webi Gestro | I4:NW:L6 | III (LRB) | COL6834.1.1 | 5,629±43 | 6,491 | 6,308 | -22.5 |
| Simbero | O6:NW:L10 | IV (LSB) | Beta-610091 | 5,670±30 | 6,551 | 6,352 | -21.6 |
| Webi Gestro | I4:NW:L4 | II (CBG lower) | COL6833.1.1 | 5,699±43 | 6,630 | 6,396 | -22.9 |
| Simbero | O6:NW:L11 | IV (LSB) | Beta-490939 | 7,290±30 | 8,173 | 8,025 | -24.2 |
| Umburi | T1:SE:L8 | V (ABM) | Beta-490937 | 8,020±30 | 9,011 | 8,770 | -22.7 |
| Gata I | P2:SW:L16 | XII (MAT) | Beta-503926 | 8,880±30 | 10,177 | 9,820 | -23.1 |
| Dimtu | C2:NE:L8 | III (DBR) | COL6830.1.1 | 12,181±57 | 14,315 | 13,861 | -22.1 |
| Webi Gestro | I4:NW:L8 | IV (SDB) | COL6835.1.1 | 12,211±58 | 14,330 | 13,882 | -22.5 |
| Webi Gestro | I4:NW:L15 | V (LSB) | COL6837.1.1 | 12256±61 | 14,805 | 14,040 | -21.1 |
| Webi Gestro | F3:SE:L10 | IV (SDB) | Beta-490940 | 12370±40 | 14,836 | 14,175 | -22.8 |
| Webi Gestro | I4:NW:L12 | IV (SDB) | COL6836.1.1 | 12351±61 | 14,842 | 14,103 | -17.2 |
| Simbero | K4:SW:L9 | V (CBO) | Beta-610094 | 12400±30 | 14,845 | 14,262 | -20.7 |
| Dimtu | C2:NE:L10 | III (DBR) | COL6831.1.1 | 12393±58 | 14,879 | 14,180 | -23.7 |
| Simbero | K4:NW:L10 | V (CBO) | Beta-552724 | 12460±40 | 14,958 | 14,322 | -23.0 |

**Table S2 Major and trace element data of the four rock samples from inner Big Boulder Moraine on the Sanetti Plateau.**

| **Rock sample** | **O**  **(%)** | **C**  **(%)** | **Na**  **(%)** | **Mg**  **(%)** | **Al**  **(%)** | **Si**  **(%)** | **P**  **(%)** | **K**  **(%)** | **Ca**  **(%)** | **Ti**  **(%)** | **Mn**  **(%)** | **Fe**  **(%)** | **B**  **(ppm)** | **Sm**  **(ppm)** | **Gd**  **(ppm)** | **U**  **(ppm)** | **Th**  **(ppm)** |
| --- | --- | --- | --- | --- | --- | --- | --- | --- | --- | --- | --- | --- | --- | --- | --- | --- | --- |
| SA10 | 48.03 | 0.68 | 4.62 | 0.68 | 9.08 | 28.80 | 0.06 | 4.33 | 1.15 | 0.37 | 0.14 | 3.62 | 5 | 5.1 | 4.1 | 1.3 | 9.4 |
| SA11 | 47.74 | 0.53 | 4.61 | 0.42 | 9.26 | 28.85 | 0.06 | 4.33 | 1.09 | 0.38 | 0.14 | 3.81 | 1 | 5.0 | 4.1 | 0.7 | 9.3 |
| SA12 | 47.37 | 0.56 | 4.67 | 0.43 | 8.98 | 28.67 | 0.06 | 4.27 | 1.15 | 0.38 | 0.14 | 3.78 | 3 | 5.8 | 4.4 | 0.9 | 9.8 |
| SA13 | 46.87 | 0.52 | 4.55 | 0.39 | 8.82 | 28.51 | 0.06 | 4.26 | 1.09 | 0.36 | 0.15 | 3.86 | 5 | 6.6 | 5.2 | 1.4 | 11.8 |

**Table S3 Results of electron microprobe analyses showing geochemical compositions.** Concentrations of the 16 samples are given in weight percent (wt.%). Sample stratigraphic proviniance is documented in **Fig. 3**.

| **Sample ID** | **Artifact category** | **Site** | **Layer** | **Excavation unit** | **SiO_2_** | SD | **TiO_2_** | SD | **ZrO_2_** | SD | **Al_2_O_3_** | SD | **Fe_2_O_3_** | SD | **MnO** | SD | **MgO** | SD | **CaO** | SD | **Na_2_O** | SD | **K_2_O** | SD | **F** | SD | **Cl** | SD | **SUM** | SD |
| --- | --- | --- | --- | --- | --- | --- | --- | --- | --- | --- | --- | --- | --- | --- | --- | --- | --- | --- | --- | --- | --- | --- | --- | --- | --- | --- | --- | --- | --- | --- |
| Bale 066 | Flake | Dimtu | Layer III | C2:SW:L6 | 62.38 | 0.19 | 0.50 | 0.03 | 0.03 | 0.03 | 15.50 | 0.05 | 6.65 | 0.12 | 0.36 | 0.01 | 0.36 | 0.01 | 0.84 | 0.01 | 10.15 | 0.21 | 5.20 | 0.05 | 0.32 | 0.05 | 0.28 | 0.01 | 102.55 | 0.24 |
| Bale 067 | Chip | Dimtu | Layer III | C2:SW:L7 | 70.93 | 0.62 | 0.21 | 0.02 | 0.29 | 0.03 | 10.34 | 0.09 | 4.50 | 0.13 | 0.29 | 0.01 | 0.02 | 0.01 | 0.14 | 0.03 | 7.70 | 0.21 | 4.47 | 0.07 | 0.56 | 0.06 | 0.54 | 0.00 | 99.98 | 0.90 |
| Bale 068 | Flake | Dimtu | Layer III | C2:SW:L8 | 62.76 | 0.22 | 0.52 | 0.02 | 0.01 | 0.02 | 15.46 | 0.06 | 6.63 | 0.12 | 0.36 | 0.02 | 0.35 | 0.01 | 0.84 | 0.02 | 10.19 | 0.21 | 5.24 | 0.07 | 0.37 | 0.02 | 0.27 | 0.00 | 103.02 | 0.31 |
| Bale 069 | Angular waste | Dimtu | Layer III | C2:SW:L9 | 72.49 | 0.24 | 0.24 | 0.03 | 0.28 | 0.03 | 10.33 | 0.06 | 4.43 | 0.08 | 0.36 | 0.03 | 0.03 | 0.01 | 0.19 | 0.01 | 7.55 | 0.15 | 4.52 | 0.04 | 0.50 | 0.03 | 0.46 | 0.01 | 101.41 | 0.22 |
| Bale 070 | Flake | Dimtu | Layer III | C3:SW:L10 | 72.51 | 0.24 | 0.25 | 0.02 | 0.32 | 0.05 | 10.07 | 0.21 | 4.63 | 0.13 | 0.30 | 0.01 | 0.02 | 0.00 | 0.18 | 0.01 | 7.73 | 0.10 | 4.39 | 0.09 | 0.57 | 0.04 | 0.57 | 0.02 | 101.54 | 0.13 |
| Bale 096 | Bladelet fragment | Simbero | Layer V | K4:SE:L9 | 72.38 | 0.43 | 0.25 | 0.02 | 0.29 | 0.03 | 10.06 | 0.12 | 4.77 | 0.50 | 0.35 | 0.04 | 0.03 | 0.00 | 0.21 | 0.06 | 7.27 | 0.61 | 4.35 | 0.56 | 0.48 | 0.12 | 0.24 | 0.05 | 100.68 | 0.62 |
| Bale 097 | Flake | Simbero | Layer V | K4:SE:L9 | 73.12 | 0.27 | 0.21 | 0.02 | 0.18 | 0.02 | 10.64 | 0.05 | 3.56 | 0.09 | 0.30 | 0.01 | 0.04 | 0.01 | 0.18 | 0.00 | 7.10 | 0.16 | 4.52 | 0.07 | 0.55 | 0.06 | 0.45 | 0.01 | 100.86 | 0.42 |
| Bale 100 | Angular waste | Simbero | Layer V | K4:SE:L10 | 72.39 | 1.18 | 0.25 | 0.07 | 0.26 | 0.11 | 10.66 | 1.81 | 4.33 | 0.69 | 0.33 | 0.09 | 0.02 | 0.01 | 0.18 | 0.04 | 7.75 | 0.43 | 4.56 | 0.23 | 0.48 | 0.21 | 0.44 | 0.12 | 101.67 | 0.31 |
| Bale 104 | Flake | Simbero | Layer V | K4:SE:L10 | 71.89 | 0.10 | 0.28 | 0.02 | 0.27 | 0.04 | 10.48 | 0.03 | 4.95 | 0.06 | 0.36 | 0.02 | 0.05 | 0.01 | 0.23 | 0.02 | 7.71 | 0.22 | 4.52 | 0.05 | 0.49 | 0.07 | 0.45 | 0.01 | 101.68 | 0.34 |
| Bale 043 | Flake | Webi Gestro | Layer IV | F3:SE:L12 | 71.45 | 0.25 | 0.24 | 0.02 | 0.48 | 0.03 | 9.93 | 0.51 | 5.02 | 0.11 | 0.32 | 0.03 | 0.03 | 0.01 | 0.18 | 0.01 | 6.13 | 0.24 | 4.46 | 0.08 | 0.44 | 0.07 | 0.43 | 0.02 | 99.11 | 0.52 |
| Bale 079 | Angular waste | Webi Gestro | Layer IV | I4:SW:L8 | 73.42 | 0.25 | 0.18 | 0.02 | 0.18 | 0.03 | 10.74 | 0.07 | 3.65 | 0.08 | 0.31 | 0.02 | 0.03 | 0.01 | 0.07 | 0.04 | 6.91 | 0.22 | 4.51 | 0.03 | 0.47 | 0.05 | 0.46 | 0.01 | 100.94 | 0.37 |
| Bale 080 | Chip | Webi Gestro | Layer IV | I4:SW:L9 | 72.69 | 0.07 | 0.25 | 0.01 | 0.29 | 0.02 | 9.57 | 0.35 | 4.53 | 0.09 | 0.38 | 0.04 | 0.03 | 0.01 | 0.18 | 0.01 | 6.85 | 0.54 | 4.44 | 0.07 | 0.48 | 0.08 | 0.47 | 0.03 | 100.14 | 0.87 |
| Bale 082 | Flake | Webi Gestro | Layer IV | I4:SW:L11 | 72.57 | 0.11 | 0.23 | 0.03 | 0.28 | 0.02 | 10.01 | 0.03 | 4.30 | 0.10 | 0.26 | 0.03 | 0.03 | 0.00 | 0.20 | 0.08 | 7.32 | 0.08 | 4.37 | 0.09 | 0.64 | 0.18 | 0.53 | 0.01 | 100.75 | 0.32 |
| Bale 083 | Angular waste | Webi Gestro | Layer V | I4:SW:L12 | 73.06 | 0.21 | 0.25 | 0.02 | 0.28 | 0.02 | 9.78 | 0.02 | 4.54 | 0.09 | 0.36 | 0.02 | 0.03 | 0.00 | 0.18 | 0.01 | 7.24 | 0.08 | 4.47 | 0.04 | 0.49 | 0.04 | 0.45 | 0.00 | 101.12 | 0.32 |
| Bale 085 | Bladelet fragment | Webi Gestro | Layer V | I4:SW:L14 | 72.65 | 0.41 | 0.22 | 0.03 | 0.27 | 0.03 | 9.99 | 0.05 | 4.39 | 0.07 | 0.29 | 0.02 | 0.03 | 0.01 | 0.32 | 0.39 | 7.28 | 0.05 | 4.37 | 0.08 | 0.63 | 0.25 | 0.52 | 0.01 | 100.97 | 0.28 |
| Bale 086 | Flake | Webi Gestro | Layer V | I4:SW:L15 | 67.57 | 0.17 | 0.52 | 0.03 | 0.03 | 0.03 | 12.41 | 0.03 | 5.33 | 0.18 | 0.32 | 0.03 | 0.30 | 0.01 | 0.37 | 0.01 | 8.36 | 0.14 | 5.06 | 0.08 | 0.31 | 0.03 | 0.24 | 0.01 | 100.82 | 0.41 |

**Table S4           Results of the Principal Component Analysis (PCA),** reporting eigenvalues, proportion of variance explained (%), and cumulative variance for each principal component.

| **PC** | **Eigenvalue** | **% variance** | **% cumulative** |
| --- | --- | --- | --- |
| **1** | 12.3579 | 50.514 | 50.514 |
| **2** | 7.27896 | 29.753 | 80.267 |
| **3** | 2.93858 | 12.012 | 92.279 |
| **4** | 1.25062 | 5.112 | 97.391 |
| **5** | 0.444038 | 1.815 | 99.206 |
| **6** | 0.157973 | 0.64573 | 99.852 |
| **7** | 0.017777 | 0.072665 | 99.924 |
| **8** | 0.00803943 | 0.032862 | 99.957 |
| **9** | 0.00477263 | 0.019509 | 99.977 |
| **10** | 0.00277978 | 0.011363 | 99.988 |
| **11** | 0.00186087 | 0.0076065 | 99.996 |
| **12** | 0.000997574 | 0.0040777 | 100 |

**Table S5           Loading matrix of the Principal Component Analysis (PCA),** showing the contribution of each oxide to all extracted components. Values exceeding 0.4 on the first three principal axes are highlighted in **bold**.

|  | **PC 1** | **PC 2** | **PC 3** | **PC 4** | **PC 5** | **PC 6** | **PC 7** | **PC 8** | **PC 9** | **PC 10** |
| --- | --- | --- | --- | --- | --- | --- | --- | --- | --- | --- |
| **SiO_2_** | -0.5959 | **0.73462** | -0.08704 | 0.28422 | 0.097673 | -0.081649 | -0.0058981 | 0.013425 | 0.01509 | 0.011033 |
| **TiO_2_** | 0.021624 | -0.015709 | -0.0002544 | -0.0066287 | 0.075969 | -0.069855 | -0.070117 | 0.083149 | 0.73587 | -0.03088 |
| **ZrO_2_** | 0.0010522 | 0.0083804 | -0.048605 | -0.0095809 | -0.034367 | -0.022259 | 0.5725 | 0.22479 | -0.095079 | -0.68077 |
| **Al_2_O_3_** | -0.17422 | -0.35343 | **0.5582** | 0.53048 | 0.43619 | -0.1689 | 0.14247 | -0.10762 | -0.022666 | 0.0067626 |
| **Fe_2_O_3_** | **0.48915** | 0.17865 | -0.47235 | 0.14928 | 0.66924 | -0.16115 | 0.044755 | -0.048188 | -0.038217 | 0.0067994 |
| **MnO** | **0.55733** | **0.52337** | **0.57881** | 0.001928 | -0.19203 | -0.20483 | 0.030947 | -0.025643 | 0.00018566 | 0.000852 |
| **MgO** | 0.0045628 | -0.023933 | 0.016839 | 0.00079233 | 0.021749 | -0.045172 | -0.017556 | 0.16985 | 0.56402 | 0.10154 |
| **CaO** | -0.0010846 | -0.040953 | 0.074357 | 0.0086161 | 0.10187 | -0.14528 | -0.25934 | 0.90939 | -0.21892 | 0.11224 |
| **Na_2_O** | 0.212 | -0.09793 | -0.29907 | 0.75874 | -0.50719 | -0.056775 | -0.11974 | 0.026941 | 0.00033624 | -0.018223 |
| **K_2_O** | 0.13748 | 0.1317 | 0.13579 | 0.19421 | 0.15641 | 0.9291 | -0.030682 | 0.12701 | 0.039712 | -0.026882 |


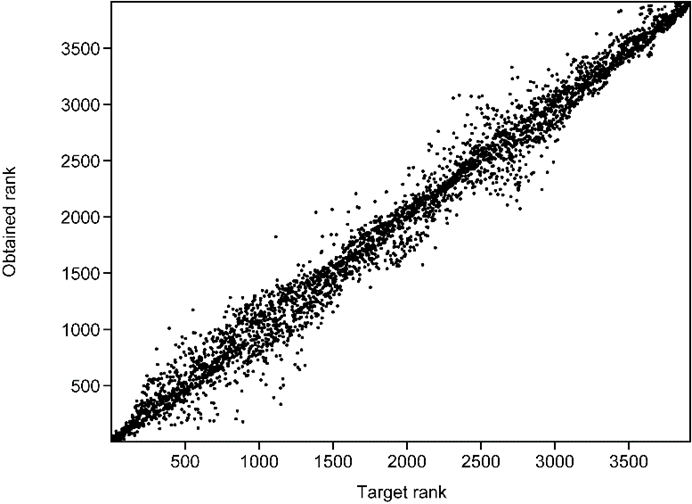


**Fig. S1 Shepard plot of the non-metrical Multidimensional Scaling (NMDS),** showing the correspondence between the original dissimilarity ranks (x-axis) and the distances in the NMDS configuration (y-axis).


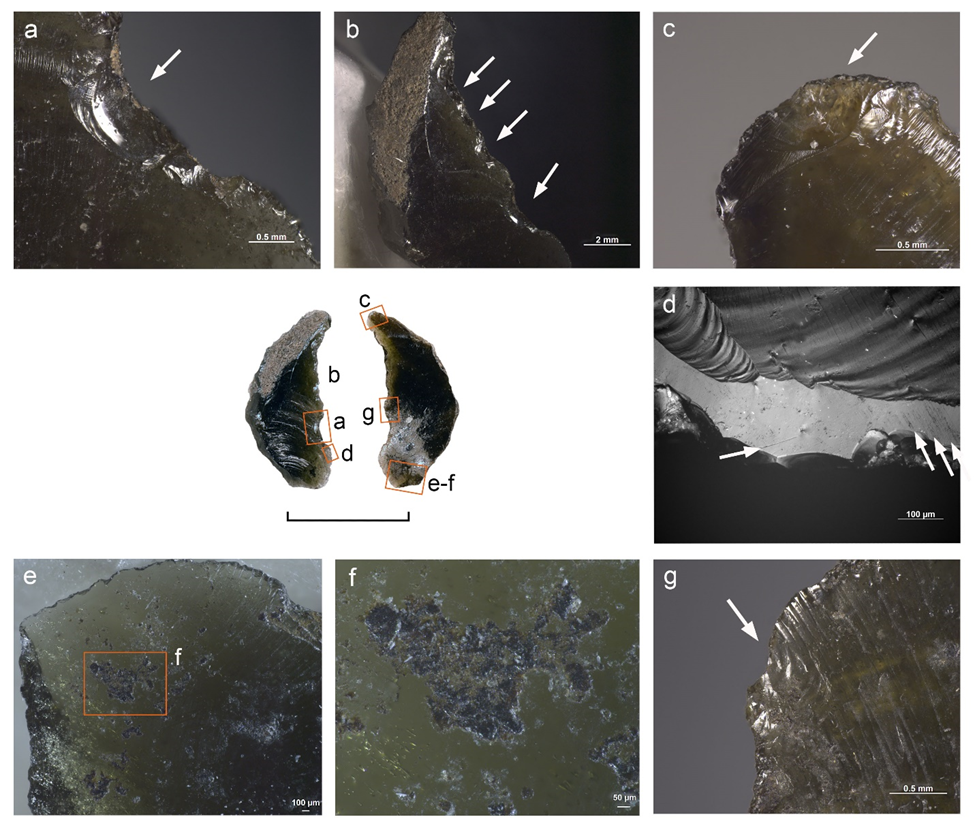


**Fig. S2              Use-wear and residues on backed piece ID 740 from Simbero. A** Bending-initiated scar with a slightly oblique orientation on the cutting edge. **B** Overview of damage in the distal portion. **C** Elongated removal at the distal extremity. **D** Linear features with oblique orientations associated with minor scarring in the proximal part. **E-F** Deposit of black residue on ventral proximal surface. **G** Obliquely oriented bending-initiated scar on ventral medial edge.


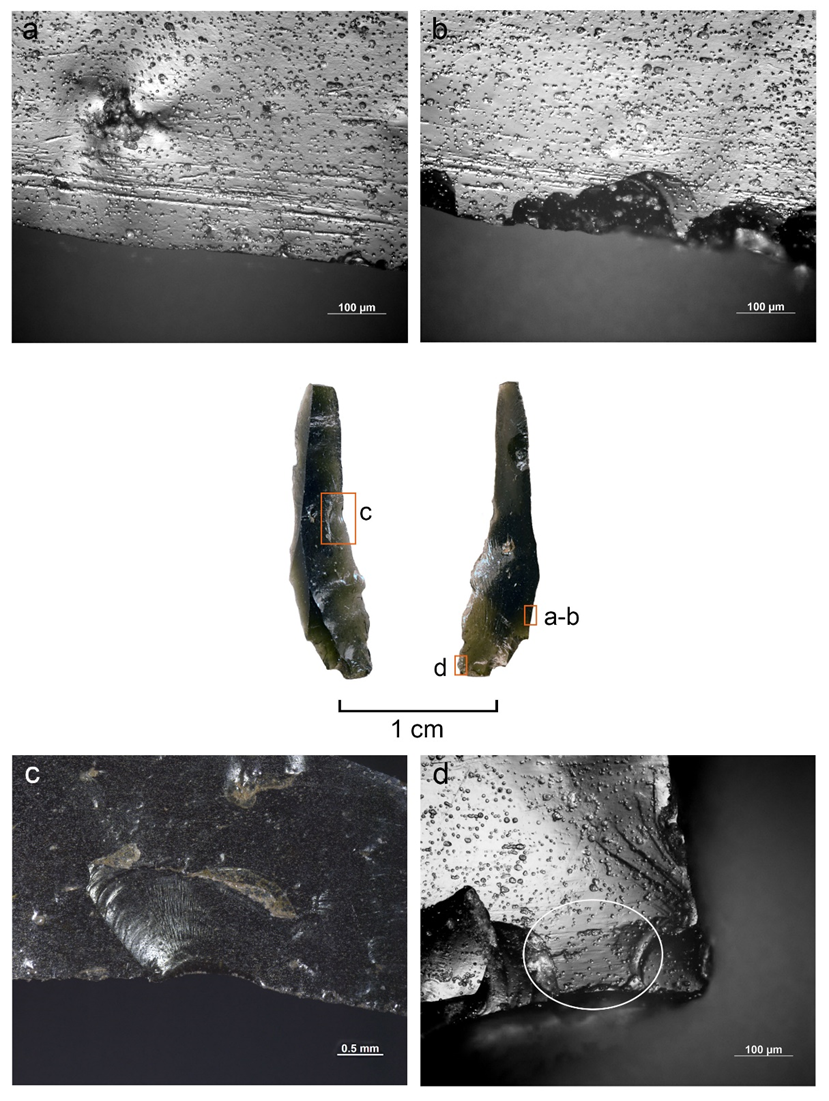


**Fig. S3 Wear features on bladelet ID 528 from Webi Gestro**. **A-B** Linear features parallel to the edge in the proximal portion of the left lateral edge. **C** Bending-initiated scar on the right edge related to hafting or use. **D** Linear wear in the proximal extremity of the right edge, possibly from hafting.


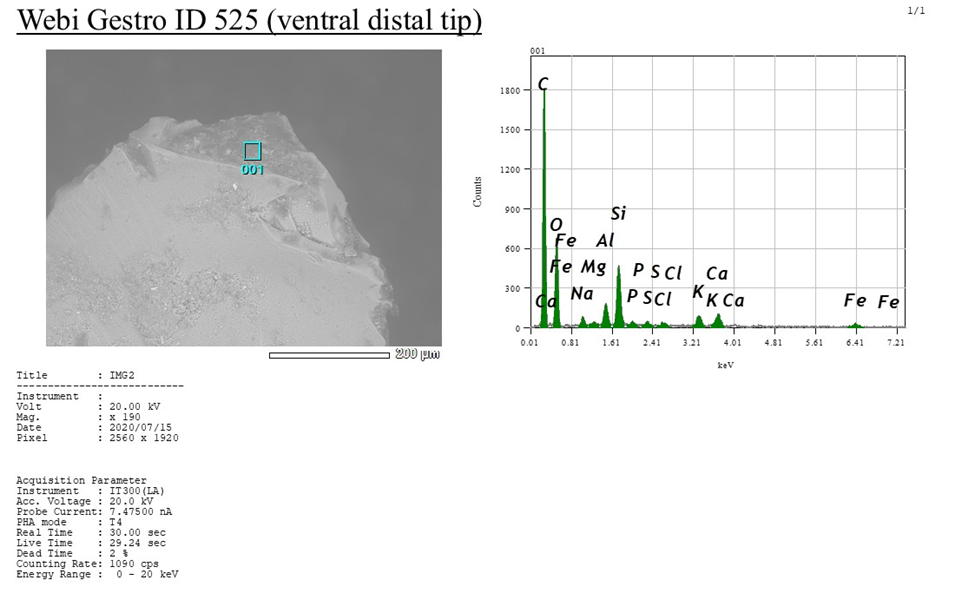


**Fig. S4 SEM-EDX spectrum of the residue observed on the ventral distal tip of ID 525 from Webi Gestro.**


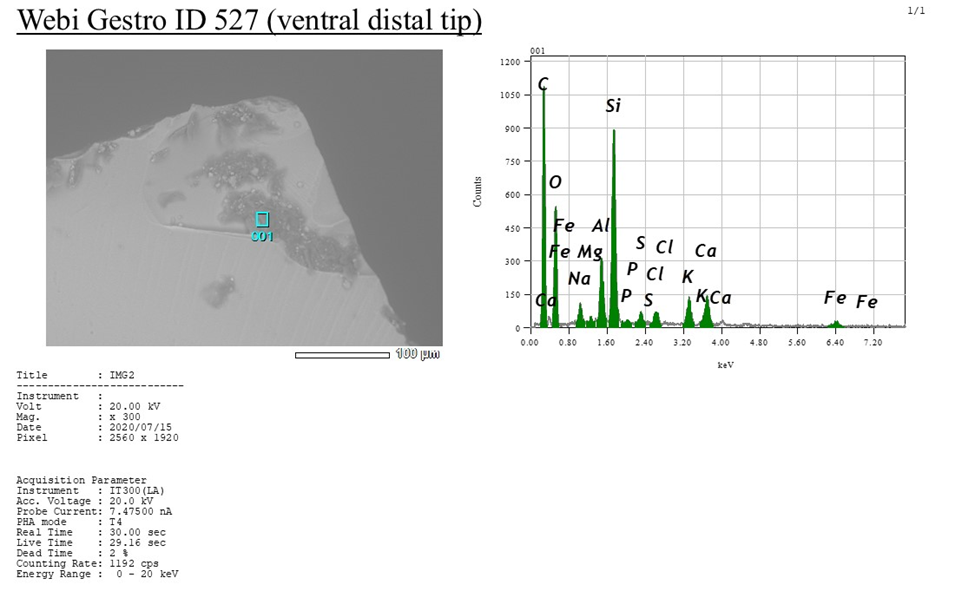


**Fig. S5 SEM-EDX spectrum of the residue observed on the ventral distal tip of ID 527 from Webi Gestro.**
